# *Blomia tropicalis* allergens induce lung DNA methylation changes in neuroimmune genes in a mouse model of airway inflammation

**DOI:** 10.3389/fimmu.2026.1775662

**Published:** 2026-07-02

**Authors:** Kevin Llinás-Caballero, Nathalie Acevedo, Simon Kebede Merid, Karen Donado, Hector Espinoza, Ernesto Mondol, Randy Reina, Ronald Regino, Inés Benedetti, Josefina Zakzuk, Leonardo Puerta, Erik Melén, Luis Caraballo

**Affiliations:** 1 Institute for Immunological Research, University of Cartagena, Cartagena, Colombia; 2 Department of Clinical Science and Education, Södersjukhuset, Karolinska Institutet, Stockholm, Sweden; 3 Departamento de Investigación e Innovación, Fundación Universitaria Antonio de Arévalo Unitecnar, Cartagena, Colombia; 4 Histopathology Research Group, School of Medicine, Universidad de Cartagena, Cartagena, Colombia

**Keywords:** allergen exposure, Blo t 13, Blo t 2, *Blomia tropicalis*, DNA methylation, house dust mite, lung inflammation, mice model

## Abstract

**Background:**

House dust mite (HDM) *Blomia tropicalis* is a major global inducer of allergic asthma, yet its epigenetic impact on airway tissues remains poorly understood. The aim of this study was to perform an exploratory, hypothesis-generating screening of the DNA methylation changes that occur in lung tissue of mice with robust allergic inflammation compared to saline-exposed controls.

**Methods:**

We sensitized and challenged BALB/c mice with *B. tropicalis* extract or purified allergens Blo t 2 and Blo t 13 or saline solution in a model of acute allergic airway inflammation, then assessed lung DNA methylation across over 285.000 CpG sites using the Infinium Mouse Methylation BeadChip (Illumina) (n = 6 mice per group, 24 lung samples).

**Results:**

Allergen-exposed mice exhibit distinct lung DNA methylation profiles compared to controls following exposure to Blo t 2, Blo t 13, or *B. tropicalis* extract. Analysis of differentially methylated regions (DMRs) revealed 137, 179 and 313 DMRs in mice exposed to Blo t 2, Blo t 13, or *B. tropicalis* extract, respectively. Genes in key differentially methylated regions included protocadherin alpha genes (*Pcdha1 to Pcdha6*), cadherin 8 (*Cdh8*) and interleukin 11 receptor alpha (*Il11ra*). Genes in DMRs were enriched in several biological processes including homophilic cell adhesion via plasma membrane adhesion molecules and nervous system development. Notably, there was also found significant differences in mRNA levels of neuronal growth genes such as (*Gdf7*), N-acetylated alpha-linked acidic dipeptidase 2 (*Naalad2*), prune homolog 2 with BCH domain (*Prune2*), cerebellin 1 precursor (*Cbln1*) and, in the proapoptotic gene *Bcl2l11.*

**Conclusion:**

Exposure to *B. tropicalis* allergens is associated with lung DNA methylation changes in genes implicated in allergic asthma and inflammatory responses, offering novel epigenetic insights and key targets of house dust mite-induced airway allergy. This exposure may involve a joint neuro-immune epigenetic reprogramming in the lung, altering the methylation of cell-adhesion and local neural signaling genes, alongside key lipid inflammatory drivers. Our results support the hypothesis that *Blomia tropicalis* exposure disrupts the airway epithelial barrier and sensitizes the local pulmonary neuroendocrine network, strongly predisposing the tissue to hyperreactivity and allergic inflammation.

## 1 Introduction

Asthma is a complex, multifactorial disease that affects approximately 260 million people worldwide (1). Among the main environmental inducers of asthma are house dust mites (HDM), such as *Dermatophagoides pteronyssinus* and *Blomia tropicalis*, which trigger several immune pathways leading to increased mucus production, bronchial hyperreactivity and bronchial remodeling (2). Despite extensive research, the pathogenesis of HDM-induced asthma remains incompletely understood, particularly regarding how allergen contact can modify gene expression and immune pathways in lung tissue (3).

Epigenetic mechanisms, such as DNA methylation, mediate the effects of environmental factors on gene expression (4). While many studies have investigated DNA methylation changes associated with asthma in humans (5), using either peripheral blood (6–9) or airway samples (10–13), only a few have examined DNA methylation changes in the airways specifically induced by HDM exposure, and those that have primarily used *Dermatophagoides* extracts (14, 15). *In vitro*, HDM exposure leads to modifications in DNA methylation and hydroxymethylation in human bronchial epithelial cells – changes thar are also observed following exposure to diesel exhaust particles or cigarette smoke – and these modifications often occur in genes involved in immune response, oxidative stress, and epithelial repair (16, 17). In mouse airways, both acute and chronic exposure to *Dermatophagoides* spp., extracts alter the DNA methylation landscape, affecting genes and pathways related to inflammation; smooth muscle contraction; and remodeling (18, 19). However, the effects of *Blomia tropicalis* extract or its individual allergens on airway DNA methylation have not yet been explored.

*B. tropicalis* is a major inducer of allergic asthma and sensitization worldwide (20), particularly in tropical regions where it is highly prevalent (21–24). To date, 14 components of this HDM have been officially recognized as allergens. Multiomics approaches have revealed that *B. tropicalis* shares 27 known or predicted allergen groups with pyroglyphid house dust mites (genus *Dermatophagoides*), and at the protein level, Blo t 2 and Blo t 13 are among the most abundant allergens (25). Blo t 2 is an ML-domain protein belonging to the MD-2-related lipid-recognition domain family, with unique epitopes and naturally occurring isoforms capable of inducing allergic sensitization in susceptible individuals (26, 27). Blo t 13 is a fatty acid binding protein (28, 29), that is recognized by the acute-phase protein serum amyloid A1, leading to the release of IL-33 and the promotion of pulmonary type 2 immunity (30). Despite their importance, the impact of *B. tropicalis* allergens on airway DNA methylation remains unknown. Since these allergens have different mechanisms of action (30, 31), we aimed to evaluate if they induce different epigenetic modifications at the beginning of the allergic response and, if there are differences in lung DNA methylation in mice exposed to purified molecules or the whole mite extract.

The mouse model of acute lung inflammation has been previously used to evaluate the immunological effects of acute allergen exposure, including epigenetic studies (18, 32). Recent evidence suggests that airway hyperreactivity is not only driven by classical immune cells but also by local neuro-immune interactions (33). Therefore, evaluating the epigenetic landscape may uncover novel neural and cell-adhesion pathways driving asthma pathogenesis. The aim of this study was to investigate the changes in lung DNA methylation after exposure to *B. tropicalis* extract, Blo t 2 and Blo t 13 in mice to explore epigenetic signatures associated with a well-defined allergic airway inflammation phenotype.

## 2 Methods

### 2.1 Study design

This study is an experimental exploratory comparison of lung inflammatory variables, DNA methylation levels and mRNA expression in 24 mice receiving house dust mite allergens (exposed group) or saline solution (control group) as an acute allergy model. The experiments were conducted in three independent cohorts, comprising a total of 62 mice distributed across saline solution, Blo t 2, Blo t 13, and B. tropicalis extract exposure groups. For genome-wide DNA methylation analyses, 24 mice (n = 6 per group) were selected based on predefined criteria of successful allergic sensitization, including allergen-specific antibody production, dose-dependent increases in Penh (enhanced pause) during methacholine challenge, increased inflammatory cell infiltration in BAL fluid, and histopathological evidence of airway inflammation (**Supplementary Figure 1**). Animals not fulfilling these criteria were excluded from methylation profiling. This selection approach was applied to ensure consistent induction of the allergic phenotype and adequate biological signal for epigenomic analyses. This study is reported in accordance with ARRIVE guidelines and, all protocols were approved by the Committee on the Ethics of Animal Experiments of University of Cartagena (Act Nr. 128, date 14/11/2019). All experiments were performed in compliance with the Guide for the Care and Use of Laboratory Animals of the Republic of Colombia (Res 8430-1993).

### 2.2 Allergens

Blo t 2 (Batch EO1004) and Blo t 13 (Batch EB1001) were produced as described previously (27, 34). *B. tropicalis* extract (Batch, Bt19) was obtained from house dust mites collected and cultured in Cartagena, Colombia, and prepared as previously described (21, 35). The quality of recombinants and extract was confirmed by 12% SDS-PAGE, and protein concentration determined by densitometry and Bradford respectively. The same recombinants and extract batches were used in all experiments. Endotoxin in the recombinant proteins and extract was removed using 1% Triton X-114 following a protocol described elsewhere (36). Endotoxin levels were determined using a quantitative colorimetric assay based on the Limulus Amebocyte Lysate (LAL) reaction (Toxin SensorTM Endotoxin Detection System, Cat. L00350. GenScript, Piscataway, NJ, USA). Mice received for each dose 20 μg of rBlo t 2 (0.029 UE/µg of protein), rBlo t 13 (0.114 UE/µg of protein), *B. tropicalis* extract (0.26 UE/µg of protein) or pyrogen-free saline solution depending on the experimental group.

### 2.3 Sensitization protocol

**Figure 1:**
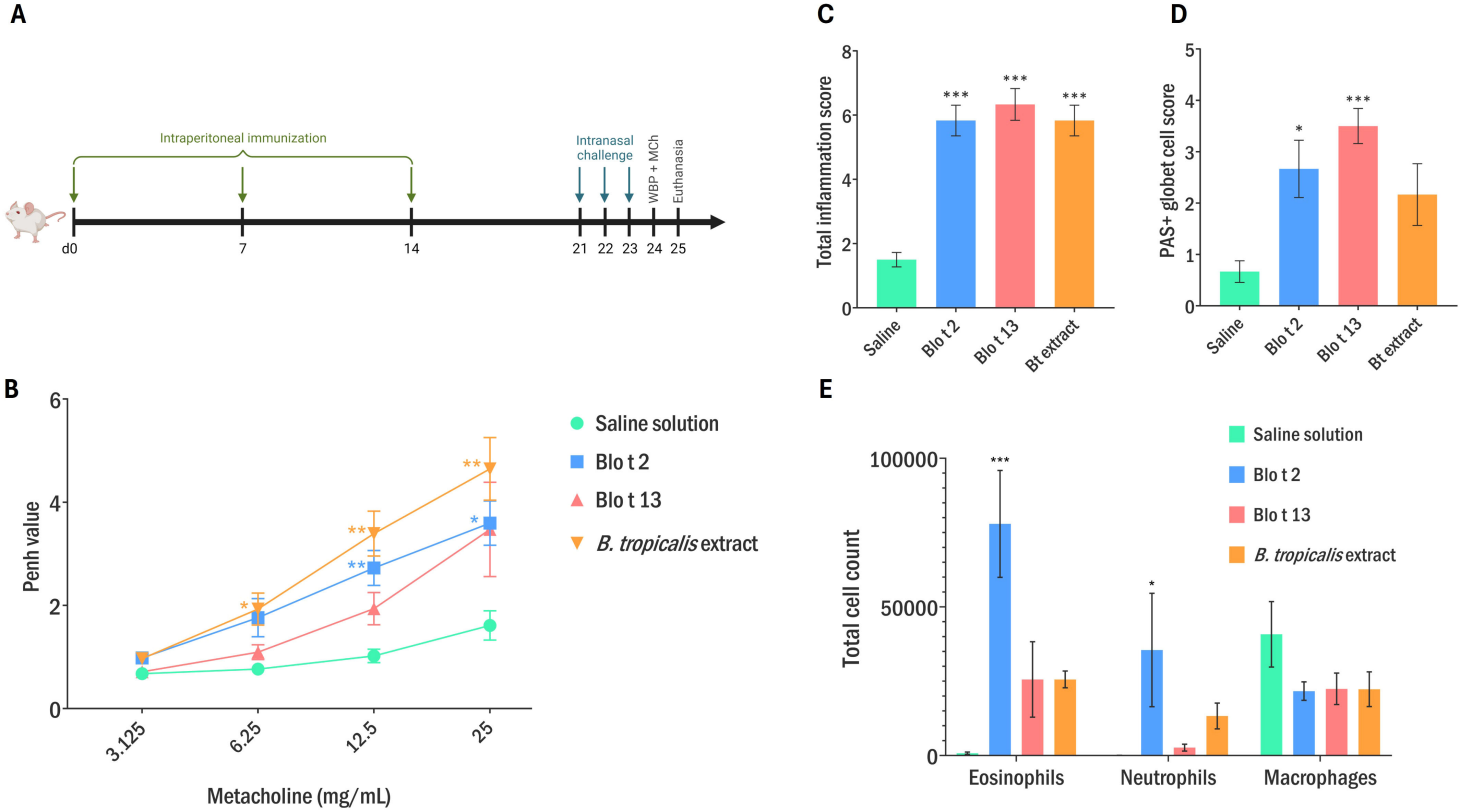
Mice exposed to *B. tropicalis* allergens exhibit features of allergic airway inflammation. **(A)** Experimental model of immunization and challenge with Blo t 2, Blo t 13, *B. tropicalis* extract or saline solution in BALB/c mice **(B)** and Penh values obtained from whole body pletismography (WBP) with methacholine challenge per each group (lower panel). **(C)** Total inflammation in lung tissue sections. **(D)** Periodic acid–Schiff positive (PAS+) goblet cell scores in lung tissue sections. **(E)** Total cell counts of eosinophils (Siglec-F^+^ Ly6G^-^), neutrophils (CD11b^+^ Ly6G^+^) and alveolar macrophages (Siglec-F^+^F4/80^+^) in bronchoalveolar lavage (BAL) fluid. Data are from six biologically independent mice per group and presented as mean ± standard error of the mean (SEM). *P < 0.05; **P < 0.01; ***P < 0.001; one-way ANOVA with Dunnet’s *post hoc* test.

Six weeks-old female BALB/c mice were obtained from the National Institute of Health (INS, Bogotá; Colombia) and randomly allocated in four subgroups of 6 animals. The groups were named after the treatment they received either saline, Blo t 2, Blo t 13 and *B. tropicalis* extract. The animals were housed in cages with a light/dark cycle of 12 hours, water supply *ad libitum* and standard diet (Rodent LabDiet Ref 5010). The exposure protocol was performed as follows: individual allergens and *B. tropicalis* extract were emulsified in Imject™ Alum (Thermo Fisher Scientific; US) and injected to mice intraperitoneally (i.p.) once a week during 3 weeks. One week later; mice were intranasally challenged once daily on three consecutive days with the same allergen used for i.p. immunization (**Figure 1A**). At day 24, whole-body plethysmography (WBP) was performed (Buxco Electronics; US) with methacholine challenge at increasing concentrations. At day 25 mice were euthanized with an intraperitoneal injection of 150 μL of sodium phenobarbital plus diphenylhydantoin (Euthanex^®^ INVET; Bogotá; Colombia).

### 2.4 Antibody measurements

Allergen specific antibody levels (IgG_1_ and IgG_2a_) in serum samples were measured by ELISA using a validated assay. A mouse polyclonal antiserum with known specificity was used as a positive control, and serum from a non-immunized mouse was used as a negative control. In addition, PBS containing 0.1% BSA was included as a blank control. Optical density (OD) was measured at 405 nm using Multiskan GO spectrophotometer (Thermo Scientific, US).

### 2.5 Bronchoalveolar lavage fluid analysis

Bronchoalveolar lavage (BAL) fluid was collected to evaluate cellularity (i.e.; eosinophil; neutrophil; and macrophage cell counts) by flow cytometry. In brief, BALF was harvested by flushing the lung airways via the trachea (2×) with 1 mL of ice-cold PBS containing a 1% complete protease inhibitor cocktail (Ref. P2714, Sigma-Aldrich, St. Louis, MO, USA). After centrifugation for 5 min at 1.500 rpm at 4 ◦C, supernatants were collected and stored at −80 °C. The cell pellet was resuspended in 1 mL of PBS and mixed with 1 mL of 1X lysis buffer (cat. 555899, BD Pharm Lyse™, Franklin Lakes, NJ, USA) it was left in incubation for 5 min on ice and then centrifuged at 1.500 rpm for 5 min. Cells were resuspended in 60 µL of Stain buffer (PBS 0.5% BSA), and 40 µL of this suspension was mixed with 10 µL of the monoclonal antibodies cocktail (V450-CD45, PECy7-CD3e, FITC-F4/80, PE-CD11b, PerCPefluor-CD170 (SiglecF), APC-Ly6G) or 10 µL of the isotype control cocktail to define the different cell populations. Stain reaction was incubated for 30 min at 4 °C in the dark and washed by centrifugation with 500 µL of Stain Buffer for 7 min at 300 g. Finally, cells were resuspended in Fixation Buffer (PBS + 0.1% formaldehyde) and the different cell populations were analyzed by flow cytometry using the FACS-ARIA III system (BD, Franklin Lakes, NJ, USA).

### 2.6 Lung samples and histology analysis

The right lung was removed and placed in 10% neutral-buffered formaldehyde for histological analysis. The samples were evaluated by a pathologist blinded for group assignments. The lung samples were embedded in paraffin and then cut into 4 µm thick sections and stained H/E (Ref. 6765015, Thermo Shandon, Pittsburgh, PA, USA) and PAS (Ref. HX99153846, Merck, Darmstadt, Germany). The sections were visualized by light microscopy to evaluate lung inflammation and mucus production. Lung inflammation was defined as the sum of the peribronchial and perivascular inflammation scores based on a modification of the 5-point scoring system described by (37). The degree of mucus production was determined by counting Periodic acid–Schiff positive (PAS+) goblet cells using the 5-point scoring system described by (38). Images were captured at a magnification of 10× and 40× on a light microscope connected to an ICC50 HD camera DM500 (Leica Microsystems, Wetzlar, Germany). Images were processed with Leica Application Suite software version 3.0.

For DNA methylation analyses; a longitudinal section of the left lung was obtained and fragmented into small pieces that were snap-frozen by submersion in an ethanol/dry ice bath and then stored at -80 °C until DNA extraction. For gene expression analyses; another section of the same lung was fragmented into small pieces and stored in RNAlater solution (Thermo Fisher Scientific; US) at -80°C until RNA extraction.

### 2.7 DNA extraction; quantification; and quality control

Mouse lung tissue samples were thawed at room temperature; immersed in PBS; and homogenized using a TissueRuptor rotor-stator homogenizer (QIAGEN; Hilden; Germany). DNA was isolated using the QIAamp DNA Mini Kit (QIAGEN; Germany) according to the manufacturer’s instructions. DNA concentration (ng/μL) and quality (absorbance ratios) were quantified by UV spectrophotometry using a NanoDrop 2000c spectrophotometer (Thermo Fisher Scientific; US). As required for the method of DNA methylation analysis; DNA concentration was also measured by fluorometry using the Qubit™ dsDNA BR Assay Kit (Invitrogen; US) in a Qubit^®^ 2.0 Fluorometer (Invitrogen; US). The DNA yield was between 4.6 and 17.1 μg (mean ± SD = 9.5 ± 4.0 μg) as measured by spectrophotometry and between 1.14 and 2 μg (mean ± SD = 1.4 ± 0.2 μg) as measured by fluorometry. The A260/A280 ratio ranged from 1.9 to 2.0 (mean ± SD = 1.93 ± 0.04); whereas the A260/A230 ratio ranged from 1.21 to 2.5 (mean ± SD = 2.1 ± 0.3). Agarose gel electrophoresis was performed to check for DNA degradation. We aliquoted DNA samples of > 500 ng at a concentration of > 10 ng/μL for methylation analyses.

### 2.8 Sample selection

A total of 24 mice were selected (n = 6 per treatment group). Mice in which a type 2 inflammatory response was induced were selected based on airway hyperreactivity results (i.e.; increasing Penh values with higher methacholine doses and difference from control mice); serum specific antibodies levels (i.e.; higher IgG_1_ levels than control mice); BAL fluid cell counts (i.e.; higher eosinophil counts than control mice); and histopathological analyses results (i.e.; higher total inflammation score; infiltration eosinophil score and PAS+ goblet cell score than control mice) as previously described (26).

### 2.9 Genome-wide assessment of lung DNA methylation

Assessment of DNA methylation levels was carried out by the Epigenomic Services from Diagenode (Belgium) using the Infinium^®^ Mouse Methylation BeadChip microarray (Illumina; US); which measures DNA methylation in over 285;000 CpG sites throughout the mouse genome with single-nucleotide resolution and representation of various genomic elements (39). For DNA methylation analyses, 24 samples (n = 6 per group) were processed on two Infinium Mouse Methylation BeadChips (12 samples per chip). Briefly; DNA was treated with sodium bisulfite using the EZ DNA Methylation Kit (Zymo Research; US); followed by whole-genome amplification; probe hybridization; and single-base extension. Finally; the microarray was scanned using the iScan™ system (Illumina; US) and IDAT files were obtained. Samples were allocated by exposure group across the two chips and distributed across rows and columns to minimize potential batch and positional effects. Potential technical variation related to chip processing was assessed by principal component analysis of M values. Leading principal components were included as covariates in the epigenome-wide association study (EWAS) models to account for residual technical and batch-related variation. The raw DNA methylation data is available in the figshare repository (DOI 10.6084/m9.figshare.29896118).

### 2.10 Methylation data analysis

Initial quality control of methylation data was performed by analyzing the intensities of the internal controls of the microarray using GenomeStudio^®^ Software 2011.1; Methylation Module v1.9 (Illumina; US). IDAT files were imported to R version 4.4.3 and preprocessed to obtain beta values using the SeSAMe package (v1.24.0) (40). Technical noise and probe quality was analyzed with the TQCD0PB pipeline, that included strain-specific masking (flags probes prone to cross-hybridization or strain-specific polymorphisms in the mouse genome), quality masking (flags probes with poor design characteristics or ambiguous genomic mapping), channel inference (infers the correct fluorescence channel for Infinium Type-I probes to correct manifest inaccuracies), dye bias correction (applies non-linear quantile interpolation to correct residual dye bias, using Type-I probes as an internal reference). Then applied a Mask reset to all probes, leaving only signal-based quality assessment for final probe exclusion. Afterwards, an Out-Of-Band Array Hybridization pOOBAH detection p-value masking assigned NA to probes whose signal intensity was indistinguishable from the out-of-band background distribution. The detection p-value threshold was applied using the package’s default parameter (p > 0.05). Finally, Noob background subtraction performed normal-exponential deconvolution background correction parameterized by out-of-band probe signals. At the probe level, we utilized listwise deletion (complete-case analysis). Any probe that was masked in at least one of the 24 samples was excluded from the dataset prior to statistical modeling. This approach ensures that the resulting linear models were based on observed data without the need for imputation. Thereby no data imputation was performed at any stage.

Beta values were converted to M values (41) using the B2M function of the ENmix package (version 1.42.0) (42, 43) and then used for principal component analysis (PCA) with the prcomp function. We performed an epigenome-wide association study (EWAS) to estimate the association of DNA methylation M values between mice exposed to allergens and negative controls using PC-adjusted robust linear regression (rlm) in the MASS R package; version 7.3-65) with the model formula for each CpG defined as Mvalue ~ allergen + PC1 + PC2. P-values were adjusted for multiple tests using the Benjamini-Hochberg method (FDR_BH). Transcription start sites within 250 kb of significant CpGs were used to identify genes nearby DMPs; employing the “two nearest genes” rule of the Genomic Regions Enrichment of Annotations Tool (rGREAT) version 2.8.0 (http://great.stanford.edu/public/html/) (44) with whole mouse genome (assembly GRCm38; UCSC mm10) as background. DMPs were also annotated using the corresponding Illumina annotation file.

### 2.11 Differentially methylated regions

To ascertain differentially methylated regions (DMRs); EWAS results were processed with the DMRcate package version 3.2.1 (45). The analysis was performed using a Gaussian kernel smoothing approach with the developers’ recommended bandwidth (λ) of 1000 bp and scaling factor of 2; together with a significance cut-off of FDR < 0.001. In addition, DMR analysis was conducted on EWAS summary statistics after genomic inflation correction applying a stricter threshold requiring a minimum of 5 CpGs per region (min.cpgs = 5), thereby excluding smaller and potentially unstable regions. Statistical inference was based on the region-level false discovery rate (FDR < 0.05) reported by DMRcate.

### 2.12 Functional annotation and pathway analysis

To elucidate the biological significance of the loci affected by the exposures, functional annotation and pathway enrichment analysis were performed on the genes associated with the Differentially Methylated Regions (DMRs). For each exposure group (Extract, Blot13, and Blot2), unique gene sets were compiled by extracting genes directly overlapping the DMRs as well as proximal regulatory targets assigned via the Genomic Regions Enrichment of Annotations Tool (GREAT). These compiled gene lists were subsequently subjected to overrepresentation analysis (ORA) and pathway analysis using ConsensusPathDB (46). GO terms or pathways containing the genes found in this study and resulting in a FDR-adjusted p-value < 0.05 were considered significantly enriched.

### 2.13 Selection of genes for expression analyses

mRNA levels were measured in selected genes as an exploratory transcriptional follow-up to assess the potential functional relevance of certain methylation signals. These genes were selected by their biological implication in significantly enriched pathways or GO biological processes.

### 2.14 RNA extraction; quantification; and quality control

RNAlater-preserved mouse lung tissue samples were thawed at room temperature. Total RNA was isolated using the RNeasy Mini Kit (QIAGEN; Germany) according to the manufacturer’s instructions. RNA concentration (ng/μL) and quality (absorbance ratios) were quantified by UV spectrophotometry using a NanoDrop 2000c spectrophotometer (Thermo Fisher Scientific; US). The RNA yield was between 5.4 μg and 25.4 μg (mean ± SD = 12.9 ± 4.6 μg). The A260/A280 ratio ranged from 1.55 to 2.08 (mean ± SD = 1.91 ± 0.16); whereas the A260/A230 ratio ranged from 0.26 to 2.15 (mean ± SD = 1.47 ± 0.56). Integrity of RNA was also verified by agarose gel electrophoresis as two bands of 28S and 18S ribosomal RNA.

### 2.15 cDNA synthesis

Total RNA was treated with 1 μL dsDNAse (Thermo Fisher Scientific; US) for genomic DNA removal. cDNA synthesis was then performed using the SuperScript™ IV First-Strand Synthesis System (Thermo Fisher Scientific; US) according to the manufacturer’s instructions; employing either 50 µM oligo d(T)20 primer or 50 ng/μL random hexamers. cDNA was diluted; aliquoted and stored at -20°C for quantitative PCR.

### 2.16 Quantitative PCR and relative expression analyses

mRNA expression levels of selected genes were measured by quantitative PCR (qPCR) using TaqMan™ gene expression assays on a QuantStudio™ 3 Real-Time PCR System (Applied Biosystems; US); using the TaqMan™ Fast Advanced Master Mix for qPCR (Applied Biosystems; US) and cDNA synthesized with random hexamers. Subsequent validation qPCR reactions were performed using the TaqMan™ Gene Expression Master Mix (Applied Biosystems; US) and cDNA synthesized with oligo d(T)20 primer. Expression of β-actin and β2 microglobulin was used as endogenous controls. Each sample was tested by duplicate and the average cycle threshold (Ct) value exported from the QuantStudio™ Design & Analysis Software (Applied Biosystems; US). The Ct value of each endogenous control was subtracted from the Ct values of the target genes to obtain the delta Ct (ΔCt). Average ΔCt values of saline solution-treated mice were then subtracted from the ΔCt value of allergen-exposed mice and the resulting number was expressed as the delta-delta Ct (ΔΔCt). Fold changes were calculated as 2-(ΔΔCt) (47). When transcripts were undetectable in one comparison group, fold-change estimation was considered unreliable and ΔCt values were reported instead. The probes for the genes of interest were FAM-labelled; for β-actin was ABY-labelled; and for β2 microglobulin was VIC-labelled; all assays included NFQ (non-fluorescent quencher). Group comparisons were performed using the Kruskal–Wallis test on normalized expression values. Multiple-testing correction across all qPCR targets evaluated (n = 19) was performed using the Benjamini–Hochberg FDR procedure. An FDR-adjusted p-value < 0.05 was considered statistically significant.

### 2.17 Quantification of GDF7 in mice bronchoalveolar lavage fluid

GDF7 levels were measured by the Mouse GDF7 ELISA Kit (Cat. MBS2500588; MyBioSource) in BALF samples from the same mice in which DNA methylation and gene expression have been measured. In brief; 100 ul of BALF was incubated overnight at 4 °C by duplicate in a precoated plate of an anti-GDF7 antibody and incubated with 100 ul biotinylated detection antibody (Ab) during 1 hour at 37 °C. Afterwards washed 3 times and revealed with HRP and substrate solution according to the manufacturer’s instructions. After adding the stop solution; the optical densities were read at 450 nm in a Multiscan Go spectrophotometer (ThermoFisher; Vantaa; Finland). The sensitivity of the method was 9.38 pg/mL and the detection range between 15.6 and 1000 pg/mL.

### 2.18 Statistical analyses

#### 2.18.1 Phenotype variables

One-way analysis of variance (ANOVA) followed by Dunnett’s multiple comparison test was performed to compare means among > 2 groups using GraphPad Prism version 9.5.1 for Windows (GraphPad Software; US); with saline solution-exposed mice as the reference group. A *P* value below 0.05 was considered significant. Spearman’s rank correlation was performed to address the relationship between cell counts and methylation and plotted using ggpubr version 0.6.0.

#### 2.18.2 DNA methylation, inflation assessment and correction

Considering that genome-wide testing across approximately 285, 000 CpGs with n = 6 per group may be sensitive to outliers, batch effects, and model miscalibration, we conducted a detailed simulation-based statistical power analysis for our EWAS design using the model: Mvalue ~ Group + PC1 + PC2 with four allergen groups. Power was evaluated using a nominal significance threshold (*P* < 0.05) for a range of M-value effect sizes, and all other CpGs were simulated as noise. These simulations indicate that large methylation differences are likely detectable, whereas smaller differences are underpowered at the given sample size. To justify the effect size thresholds used in our analyses, we translated M-value differences into delta-beta values, providing a biologically interpretable measure of methylation change. Additionally, incorporated technical covariates (PC1 and PC2) to account for unmeasured confounding, further reducing potential miscalibration. The first two principal components of the methylation data were included to capture major sources of variation while avoiding overfitting, with the number of PCs chosen based on scree plots and the cumulative proportion of variance explained (~30–40%). PCA was performed on the normalized beta values after probe filtering to remove low-quality probes. We verified that the PCs included capturing major technical variation as intended. A fully reproducible R script with the code for EWAS, including PCA calculation, robust regression fitting, and covariate inclusion is included in the **Supplementary Material**.

Genomic inflation factors (λ lambda values) were estimated using both the PC-adjusted rlm and the Bayesian correction (Bacon) by implementing the bacon R package. This estimates the bias and inflation by comparing the empirical null distribution to a theoretical null distribution using a Bayesian framework. It generates Bacon-corrected effect sizes, standard errors, and FDR p-values (48). For this study a Bacon-corrected FDR p-value < 0.05 was considered statistically significant.

## 3 Results

### 3.1 *Blomia tropicalis* extract and individual allergens induce allergic airway inflammation

**Figure 2:**
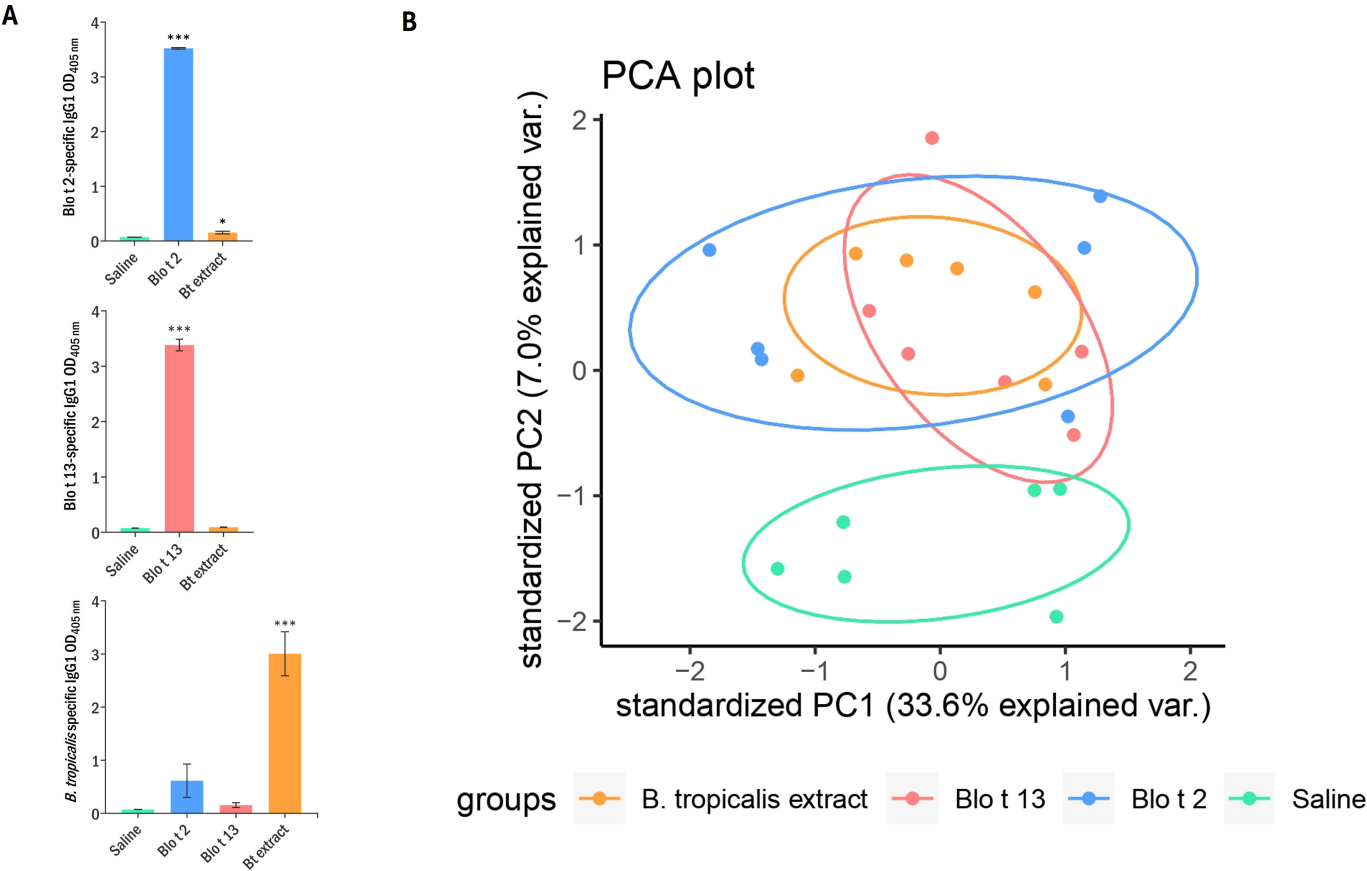
Mice exposed to *B. tropicalis* allergens developed specific antibodies to the sensitizing allergens and showed differential DNA methylation upon *B. tropicalis* exposure **(A)** Specific IgG_1_ antibody levels are shown as mean ± SEM. *P < 0.05; **P < 0.01; ***P < 0.001; one-way ANOVA with Dunnet’s *post hoc* test. **(B)** PCA plot of lung DNA methylation in mice exposed to *B. tropicalis* extract and purified allergens. Normal data ellipses are represented for each group. Data are from six biologically independent mice per group.

The sensitization protocol was performed in three independent experimental cohorts as indicated in **Figure 1A** using saline-treated mice as negative controls. Data on whole body plethysmography, allergen-specific antibodies, cell counts in BAL, and histopathological analyses were analyzed in all animals to verify successful induction of allergic airway inflammation. To enhance biological signal detection in this exploratory epigenetic screening, we implemented a phenotype-enriched sample selection approach resulting in 24 samples (6 mice per exposure group) coming from three independent mice models. We confirmed that mice exposed to Blo t 2, Blo t 13, or *B. tropicalis* extract exhibited higher Penh values in response to methacholine challenge at doses of 6.25–25 mg/mL reflecting airway obstruction and increased bronchoconstriction (narrowing); however, statistically significant differences in enhanced pause (Penh)only were only obtained in the Blo t 2- and the *B. tropicalis* extract-exposed groups (**Figure 1B**). Lung tissue sections from allergen-exposed mice also showed marked inflammation (**Figure 1C**) and mucus hypersecretion (**Figure 1D**). Analysis of BAL cell composition revealed substantial infiltration of eosinophils and, to a lesser extent, neutrophils (**Figure 1E**). Specific IgG_1_ levels, a marker of type-2 immune response in mice, were also elevated following allergen exposure confirming that Blo t 2, Blo t 13, and *B. tropicalis* extract induced specific sensitization and allergic airway inflammation in these mice (**Figure 2A**).

### 3.2 Exposure to Blo t 2; Blo t 13; or *B. tropicalis* extract modifies the mouse lung methylome

DNA was extracted from frozen lungs and global DNA methylation were measured by a microarray-based assay that evaluated over 285.000 CpG sites across the mouse genome. All samples passed quality control checks (**Supplementary Figure 2****).** At the sample level, quality was assessed by the total number of masked probes after the TQCD0PB pipeline. From the initial 296, 070 probes, 11, 688 probes (3.9%) were identified as masked across one or more samples and were subsequently removed. All 24 samples demonstrated high performance, with an average of 10, 070 probes masked per sample (approx. 3.4%). A QC flow table showing the number of probes removed or masked at each step is presented in **Supplementary Table 1**. The final analysis was performed on a filtered matrix of 284, 382 high-quality probes. M values were obtained after preprocessing the raw methylation data (**Supplementary Figure 3**, **S4**), and the beta to M value transformation did not result in further probe loss.

**Figure 3:**
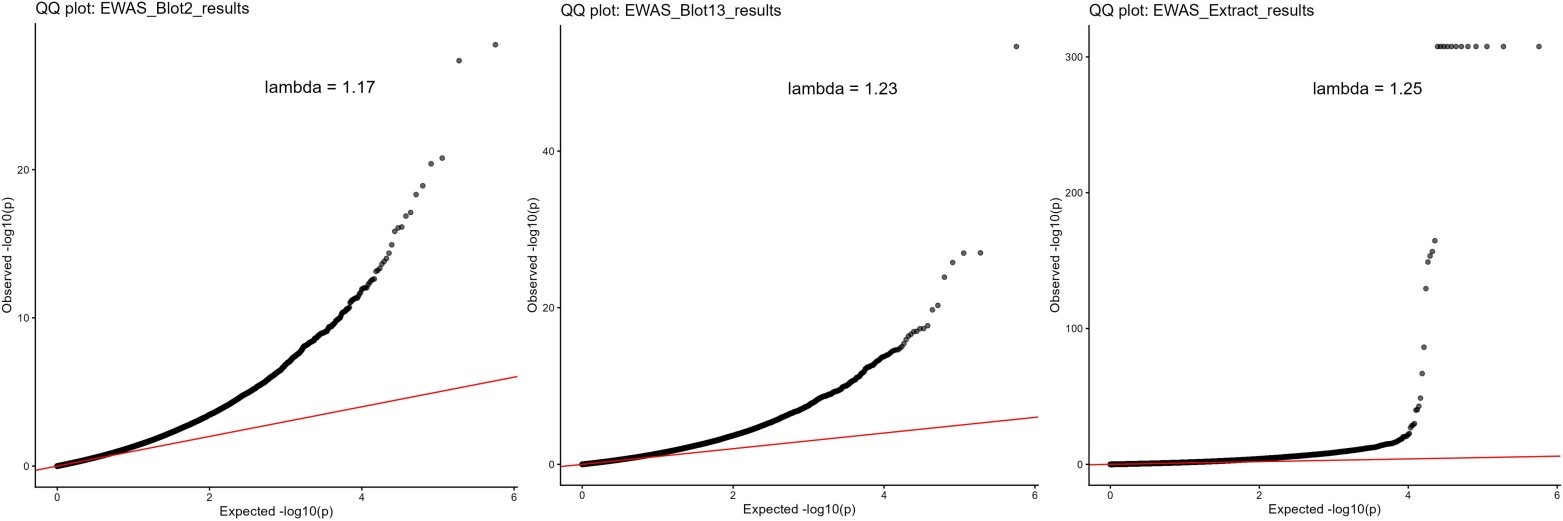
Quantile-quantile (QQ) plot for CpG-site association in the epigenome-wide association study for Blo t 2, Blo t 13 and *Blomia tropicalis* extract. The plot shows the observed -log_10_ (*P* values) (y-axis) against the expected -log_10_ (*P* values) (x-axis) under the null hypothesis of no association. The diagonal line indicates the expected null distribution. The genomic inflation factors (lambda λ) are presented for each comparison.

Principal component analysis (PCA) revealed that the DNA methylation profiles of allergen-exposed mice clustered separately from those of control mice (**Figure 2B**), indicating that these allergens may induce changes in the lung methylome. We then performed an epigenome-wide association study (EWAS) to identify differentially methylated positions in mice exposed to *B. tropicalis* extract or individual allergens compared to negative controls. A statistical power curve for differentially methylated probes is presented in **Supplementary Figure 5**. To account for genomic inflation (due to confounders like cell-type heterogeneity, batch effects, or hidden structure) that may cause excessive false-positive associations between DNA methylation and allergen exposures, we implemented the Bacon correction and the lambda value was calculated for each exposure (**Figure 3**). The genomic inflation before and after correction is presented in **Supplementary Table 2**.

### 3.3 Differentially methylated regions

**Figure 4:**
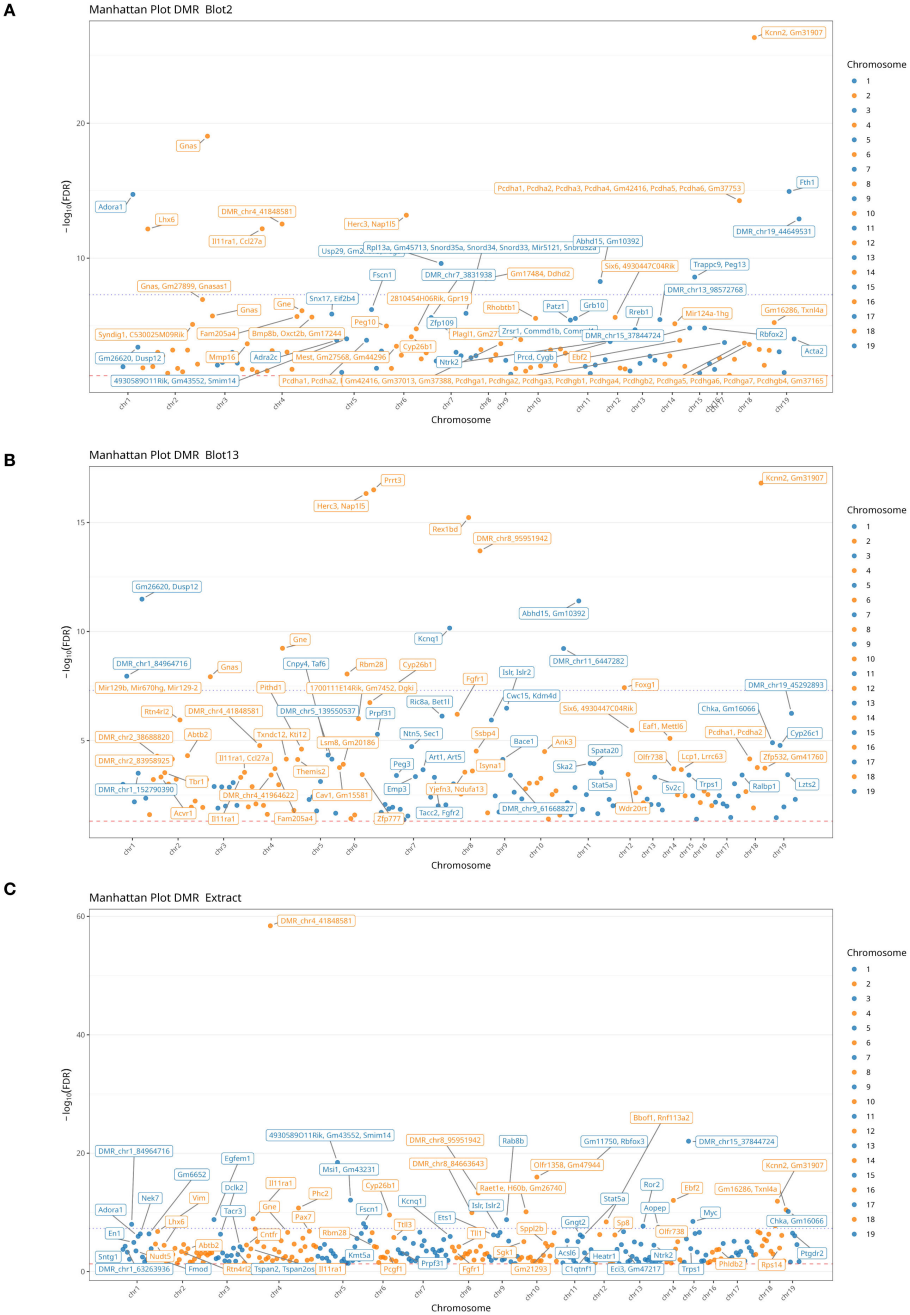
Manhattan plot of differentially methylated regions (DMRs) after allergen exposure. The genome-wide distribution and statistical significance of DMRs across chromosomes 1 through 19 is presented in **(A)** Blo t 2 **(B)** Blo t 13 and **(C)** *B tropicalis* extract. Each plotted point represents a single DMR. The points alternate between blue and orange to visually distinguish adjacent chromosomes from one another. The horizontal lines (the lower red dashed line and the higher blue dotted line) represent the conventional (P<0.05) and the genomewide statistical significance thresholds. The most highly significant DMRs are annotated with corresponding gene names (e.g., Gnas, Kcnn2, Fth1) or specific DMR positional identifiers.

Prior to running DMRcate a probe was required to have a valid detection p-value (p < 0.05) across all 24 samples (0% allowable missingness). Analysis of differentially methylated regions (DMRs) revealed 137, 179 and 313 DMRs in mice exposed to Blo t 2, Blo t 13, or *B. tropicalis* extract, respectively (**Table 1**). Detailed information on DMRs for each allergen exposure after bacon correction are presented in **Supplementary Table 3**, **S4**, **S5**. It is worth to highlight that a region in chromosome 18 showed a consistent and significant decrease in DNA methylation levels in allergen exposed mice (Blo t 2; Blo t 13 and whole *Blomia* extract) compared to saline controls. This region contained the genes encoding protocadherin alpha (*Pcdha1* to *Pcdha6*). In chromosome 4 there was also an extense DMR in the gene encoding interleukin 11 receptor subunit alpha (*Il11ra*) that was found in all the allergen exposed mice (**Table 2**). In contrast there was a region in chromosome 2 with increased methylation in allergen exposed mice within the gene encoding dual oxidase 2 (*Duoxa2*). Top DMRs between allergen-exposed mice and saline controls after bacon correction are presented in **Figures 4A–C** and **Table 2**. Exposure to *B. tropicalis* extract elicited the broadest epigenetic response, in addition to neurodevelopmental genes, there were also differences in DNA methylation levels in genes encoding solute carriers (Slc family) and metabolic transporters. Exposure to Blo t 13 and Blo t 2 produced a much more targeted epigenetic footprint that converge on the protocadherin clusters and zinc finger proteins (Zfp family). Several immune genes were found also found in DMRs (**Table 3**).

**Table 1:** A summary on the number of differentially methylated regions (DMR) between allergen-exposed mice compared to saline controls per each allergen (FDR < 0.05).

Variable	Blo t 2	Blo t 13	Bt extract
Number of DMRs	137	179	313
Mean number of CpGs	9.6	9.1	8.1
DMR size (in base pairs, bp)	924 bp	809 bp	852 bp
Min FDR	4.05 x 10^-27^	2.75 x 10^-17^	1.15 x 10^-59^
Number of DMRs (increased methylation)	89 (65%)	115 (64%)	188 (60%)
Number of DMRs (decreased methylation)	48 (35%)	64 (36%)	125 (40%)
Number of genes mapped to hypomethylated regions	130	154	284
Number of DMRs found at promoters	78 (57%)	103 (58%)	201 (64%)

The complete lists of DMR after bacon correction per each allergen are presented in the Supplementary file.

**Table 2:** Top significant DMR regions after exposure to *B. tropicalis* allergens in mice lung.

Region	Genes	Exposures	FDR	Finding
chr4:41848581-41849028	*Gm21541 Il11ra1*	Bt extract Blo t 2	3.9 x 10^-59^ 2.9 x 10^-13^	Reduced methylation
chr18:45559398-45561302	*Kcnn2*, *Gm31907 Trim36*	Blo t 2 Blo t 13	4.5 x10^-27^ 1.5 x 10^-17^	Increased methylation
chr8:95951942-95952609	*Got2* *Cdh8*	Extract Blo t 13 Blo t 2	5.1 x 10^-14^ 2.0 x 10^-14^ 2.1 x 10^-4^	Most pronounced reduced methylation in a region of 5 CpG sites
chr6:58906085-58907633	*Herc3*	Blo t 13 Blo t 2	4.1 x 10^-17^ 6.4 x 10^-14^	A region showing increased methylation
chr15:37844724-37847254	*Ncald* *Rrm2b*	Extract	9.3 x 10^-23^	Reduced methylation in 11 CpG sites
chr18:36967565-36969291	*Pcdha Gm37013*	Blo t 2	5.4 x 10^-15^	Reduced methylation (-0.44 mean difference) in the protocadherin gene family (*Pcdha1* to *Pcdha6*)
chr2:174298371-174301063	*Nelfcd*	Blo t 2	9.1 x10^-20^	This region includes 22 CpG sites with increase methylation in exposed mice

Gm: stands for gene model. In mouse genetics, this prefix is given to DNA sequences that computational algorithms predict to be genes, but which haven’t been fully characterized or named yet. *Il11ra1*: interleukin 11 receptor subunit alpha 1; *Kcnn2*: potassium calcium-activated channel subfamily N member 2; *Trim36*: tripartite motif containing 36; *Got2*: glutamic-oxaloacetic transaminase 2; *Cdh8*: Cadherin 8; *Herc3*: HECT and RLD domain containing E3 ubiquitin protein ligase 3; *Ncald*: neurocalcin delta; *Rrm2b*: ribonucleotide reductase regulatory TP53 inducible subunit M2B; *Pcdha*: protocadherin alpha cluster; *Nelfcd*: negative elongation factor complex member C/D. FDR: false discovery rate.

**Table 3:** Key immune genes found in DMR with reduced methylation in mice lung after exposure to *B. tropicalis* allergens.

Gene	Gene name	Finding	Possible effect on allergic response
*Ptgfr*	Prostaglandin F2-alpha receptor	Shows a decrease in DNA methylation in the Blot13 exposed mice affecting 9 CpG sites at region chr3:152092422-152093259. FDR = 0.009	Overexpression of this receptor is strongly linked to bronchoconstriction in allergic asthma and airway inflammation
*Alox5ap*	5-Lipoxygenase Activating Protein	Decreased methylation after all 3 exposures (down to -0.207 in the Bt. extract exposed mice) at region chr5:149287194-149287756. FDR = 0.000006 for the extract	It is essential for the synthesis of leukotrienes. It promotes eosinophil migration and inflammation in allergies and asthma.
*Ptafr*	Platelet-Activating Factor Receptor	Decreased methylation in the Bt. exposed mice at region chr4:132638828-132639151. FDR = 0.0002	Platelet-activating factor (PAF) is a critical lipid mediator in anaphylaxis, bronchial hyperreactivity, and mast cell degranulation during allergic responses.
*Stat5a and Stat5b*	Signal Transducer and Activator of Transcription 5A and 5B	Decreased methylation across all three exposure groups at region chr11:100859046-100859719. FDR = 0.000001 for the Bt. extract	Critical transcription factors for cytokine signaling
*Bcl6*	B cell lymphoma 6	Decreased methylation in Blo t 13 and Bt extract exposed mice at region chr16:23983156-23983497. FDR = 0.002 for Blo t 13.	Master transcription factor involved in antibody production, T cell function and regulating inflammation.
*Themis2*	Thymocyte selection-associated family member 2	Decreased methylation in all three exposures (mean difference of -0.28) at region chr4:132795558-132796434, FDR = 0.000002 for the Bt. extract.	Signaling adaptor protein and master regulator expressed in B cells and macrophages.
*Rhoh*	Ras homolog family member H	Decreased methylation in Bt. extract exposed mice at region chr5:65949922-65951712, FDR = 0.000000103.	RhoH belongs to the Rho family of GTPases. Involved in T cell receptor (TCR) signaling and mast cell degranulation.

### 3.4 Enrichment analysis of differentially methylated genes

**Figure 5:**
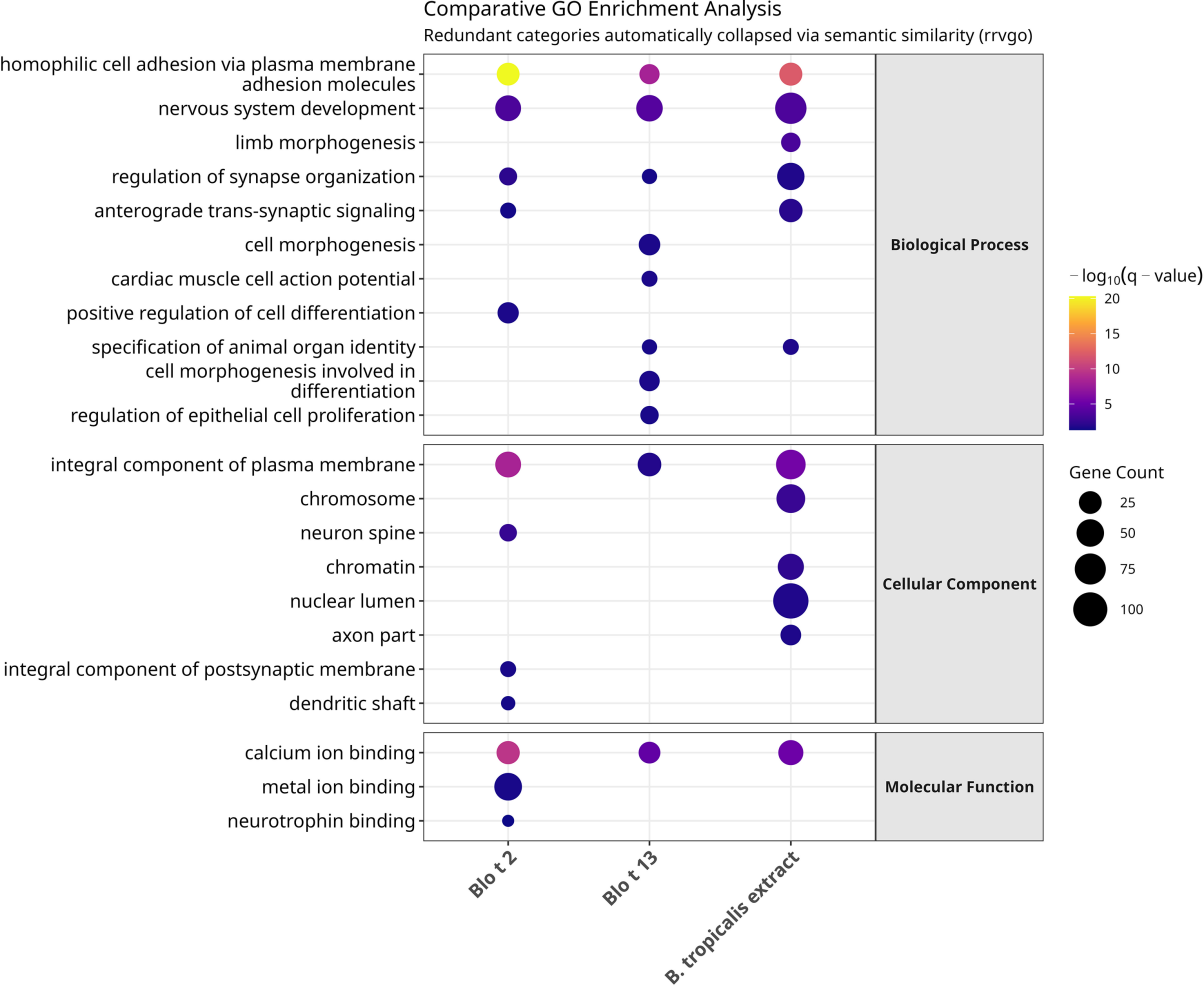
Comparative gene ontology (GO) enrichment analysis. Dot plot illustrating significantly enriched GO terms across three experimental groups: Blo t 2, Blo t 13, and *B. tropicalis* extract. To simplify the visualization, redundant GO categories were automatically collapsed based on semantic similarity using the rrvgo package. The plot is divided into three panels representing the major GO domains: biological process (top), cellular component (middle), and molecular function (bottom). The size of each dot corresponds to the gene count (the number of genes associated with that specific GO term). The color gradient reflects the statistical significance of the enrichment, calculated as the -log10(q-value); lighter yellow colors denote higher statistical significance, while darker purple colors denote lower significance.

Gene names overlapping DMRs were extracted and the list analyzed for enrichment. It is worth to note that about 12% of annotated genes were common for the three allergenic exposures. While each exposure has its own unique subset of altered genes, there was a highly conserved core of 110 genes that undergo significant methylation changes across all three datasets. This points to a specific, targeted biological effect rather than random epigenetic drift. Functional annotation revealed significant enrichment in the biological processes of cell adhesion and neuronal system development (**Figure 5**), suggesting that allergen exposure may influence these pathways. Additionally, we found significant enrichment in pathways related to nerve growth factor signaling, neutrophin response and neuroinflammation (**Tables 4**, **5**). Genes involved in cell cycle, growth, and immune signaling (e.g. *Stat5a*, *Stat5b*, *Malt1*, *Cdk1*, *Wee1*) were also enriched in DMRs. Based on the analysis of differential methylation, functional annotation and biological plausibility we then analyzed the relationship between DNA methylation and gene expression in a subset of candidate genes.

**Table 4:** Pathways associated with genes located in DMRs after Blo t 13 exposure.

Pathway	Genes in pathway	FDR
IL-7 signaling	*Cdk1, Amhr2, Stat5b, Met, Stat5a, Gfra1, Wee1, Fgfr2, Fgfr1*	0.0005
Signaling by FGFR in disease	*Stat5b, Nras, Stat5a, Fgfr2, Fgfr1*	0.0007
FGF signaling pathway	*Stat5b, Cttn, Fgfr2, Met, Fgfr1*	0.001
JAK STAT pathway and regulation	*Met, Amhr2, Nras, Stat5b, Cdk1, Stat5a, Gfra1, Wee1, Fgfr2, Fgfr1*	0.002
VEGF	*Cdk1, Amhr2, Met, Gfra1, Wee1, Fgfr2, Fgfr1*	0.008
Pathways Regulating Hippo Signaling	*Met, Fgfr1, Fgfr2, Cdh8, Gnas*	0.009
Hippo-Merlin Signaling Dysregulation	*Fgfr2, Met, Nras, Cdh8, Fgfr1*	0.01
MAPK1/MAPK3 signaling	*Cdk1, Nras, Met, Gfra1, Fgfr1, Fgfr2, Ppp2r5c*	0.01

**Table 5:** Pathways associated with genes in DMRs after exposure to *B. tropicalis* extract.

Pathway	Genes in pathway	FDR
Brain-derived neurotrophic factor (BDNF) signaling pathway	*Dlg1, Ncf2, Map2k5, Ntrk1, Ntrk2, Stat5b, Stat5a, Kcnn2, Rab3a, Rasgrf1, Bdnf, Shc1, Rack1*	0.0004
IL3-mediated signaling events	*Shc1, Pim1, Id1, Stat5b, Stat5a, Il3*	0.0008
Signaling by NTRKs	*Shc1, Sgk1, Vgf, Ntrk1, Ntrk2, Id1, Clta, Map2k5, Mef2d, Dusp7, Bdnf*	0.0013
Signaling by NTRK1 (TRKA)	*Shc1, Sgk1, Vgf, Ntrk1, Ntrk2, Id1, Clta, Map2k5, Mef2d, Dusp7*	0.0020
Signal Transduction	*Ncf2, Dlk1, Cenpn, Gngt2, Tshb, Fgfr1, Dync1h1, Gpr176, Clta, Trpc7, Map2k5, Fgd2, Bdnf, Id1, Cav1, Dlg1, Sstr1, Ptgdr2, Cdk1, Tacr3, Amhr2, Ntrk1, Ror2, Adra2c, Cyp26b1, Dgki, Pebp1, Phc2, Stat5b, Myc, Taok3, Rgs8, Vgf, Il3, Plppr5, Shc1, Gfod1, Calcrl, H2ac6, Cenpe, Npffr2, Arpc5, Syde1, Sall4, Eps15l1, Rhobtb1, Gabrb1, Pde4c, Fgfr2, Dusp7, Rack1, Sfrp2, Actn2, Tax1bp3, Tas1r2, Rasgrf1, Sgk1, Ntrk2, Vim, Spen, Met, Stat5a, Hecw1, Csnk1e, Gng3, Mef2d, Ptafr, Col6a2, Csnk2b, Ppp2r5c*	0.0020
role of erk5 in neuronal survival pathway	*Bdnf, Mef2d, Shc1, Map2k5, Ntrk1*	0.0035
Signaling by Receptor Tyrosine Kinases	*Eps15l1, Ncf2, Shc1, Sgk1, Stat5b, Vgf, Ntrk1, Ntrk2, Id1, Met, Stat5a, Clta, Map2k5, Mef2d, Gabrb1, Bdnf, Col6a2, Fgfr2, Dusp7, Fgfr1, Cav1*	0.0035
IL-7 signaling	*Cdk1, Mos, Amhr2, Ntrk1, Stat5b, Met, Stat5a, Csnk1e, Map2k5, Wee1, Fgfr2, Fgfr1*	0.0035
EPO signaling	*Cdk1, Mos, Amhr2, Ntrk1, Stat5b, Met, Stat5a, Csnk1e, Map2k5, Wee1, Fgfr2, Fgfr1*	0.0035
Hippo-Merlin Signaling Dysregulation	*Cdh12, Cdh8, Cdh18, Ntrk1, Ntrk2, Met, Myc, Fgfr2, Fgfr1*	0.0068
Pathways Regulating Hippo Signaling	*Cdh12, Cdh8, Cdh18, Ntrk1, Ntrk2, Met, Fgfr2, Fgfr1*	0.0074
mBDNF and proBDNF regulation of GABA neurotransmission	*Gabrb1, Bdnf, Shc1, Ntrk2, Gabra4*	0.0086
Ras signaling	*Rasgrf1, Ntrk1, Ntrk2, Gng3, Met, Ets1, Gngt2, Pla2g1b, Fgfr2, Shc1, Fgfr1*	0.0086

### 3.5 Neuronal related genes were enriched in DMR of allergen exposed mice

**Figure 6:**
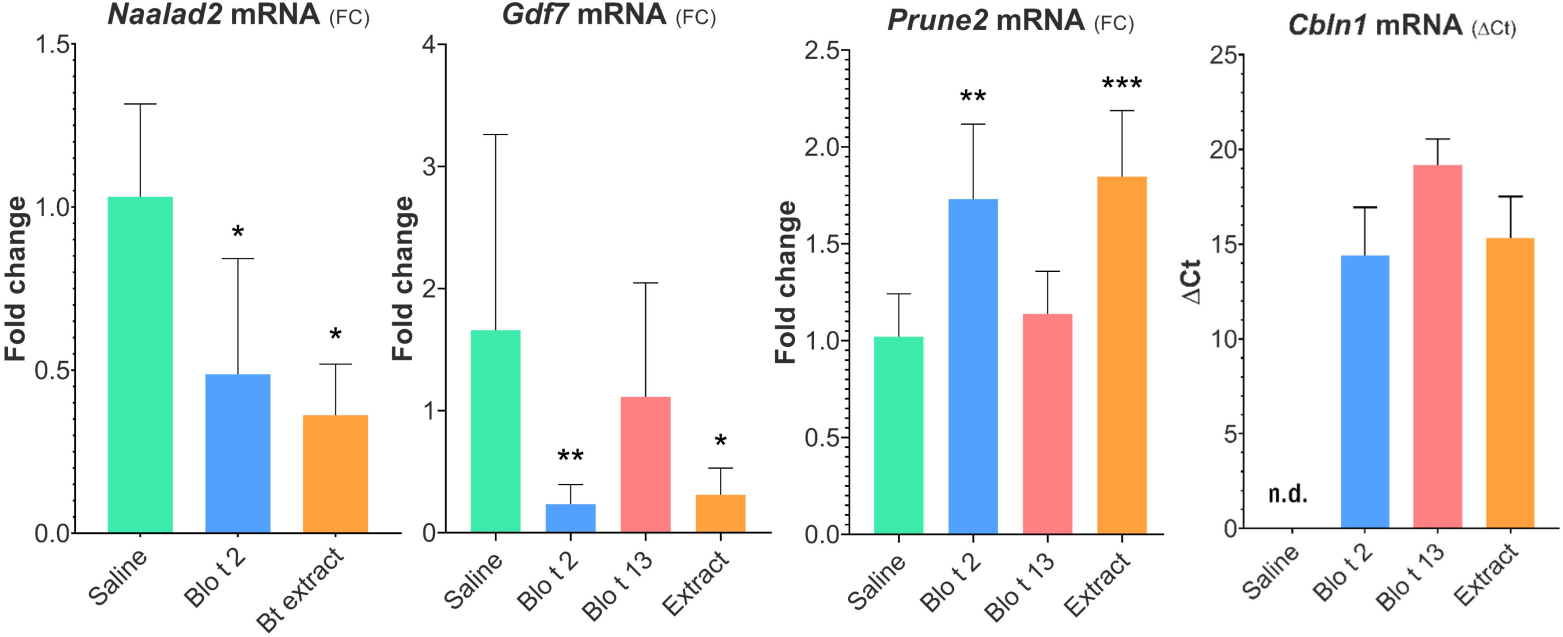
Relative mRNA expression of neuron related genes in mouse lungs using β2 microglobulin as endogenous control. *p < 0.05, ** P < 0.01, *** P < 0.001; one-way ANOVA with Dunnet’s *post hoc* test of ΔCt values. Data are from six biologically independent mice per group. *Naalad2, Gdf7*, *Prune2* and *Cbln1*. Note that mRNA expression here is expressed as ΔCt since fold change values could not be calculated.

Several DMRs were found within genes involved with brain derived neurotrophic factor (BDNF), neurotrophin receptor binding, and neuron development. We evaluated mRNA levels of neuronal related genes in mouse lung tissue (**Figure 6**). We found significantly lower expression of *Naalad2* and *Gdf7* in mice treated with Blo t 2 or *B. tropicalis* extract while on the other hand, there were increased mRNA levels of *Prune2* in the lung tissue of mice exposed to Blo t 2 and the *Blomia* tropicalis extract but not in the control group. The exposure to *Blomia* allergens also increases the expression of cerebellin 1 (*Cbln1*) in a tissue where it is not normally expressed (**Figure 6**). This finding was verified using cDNA synthesized with oligo d(T)20 primer to enrich for coding transcripts, confirming that only in allergen exposed mice there were detectable transcripts of cerebellin 1 (**Supplementary Figure 6**). Ct values for endogenous controls were found stable among different experimental groups (**Supplementary Table S6**; **Supplementary Figure 7**) and differences in gene expression were found significant after correction for multiple testing.

### 3.6 Lung DNA methylation in allergen-exposed mice was associated with eosinophil cell counts

**Figure 7:**
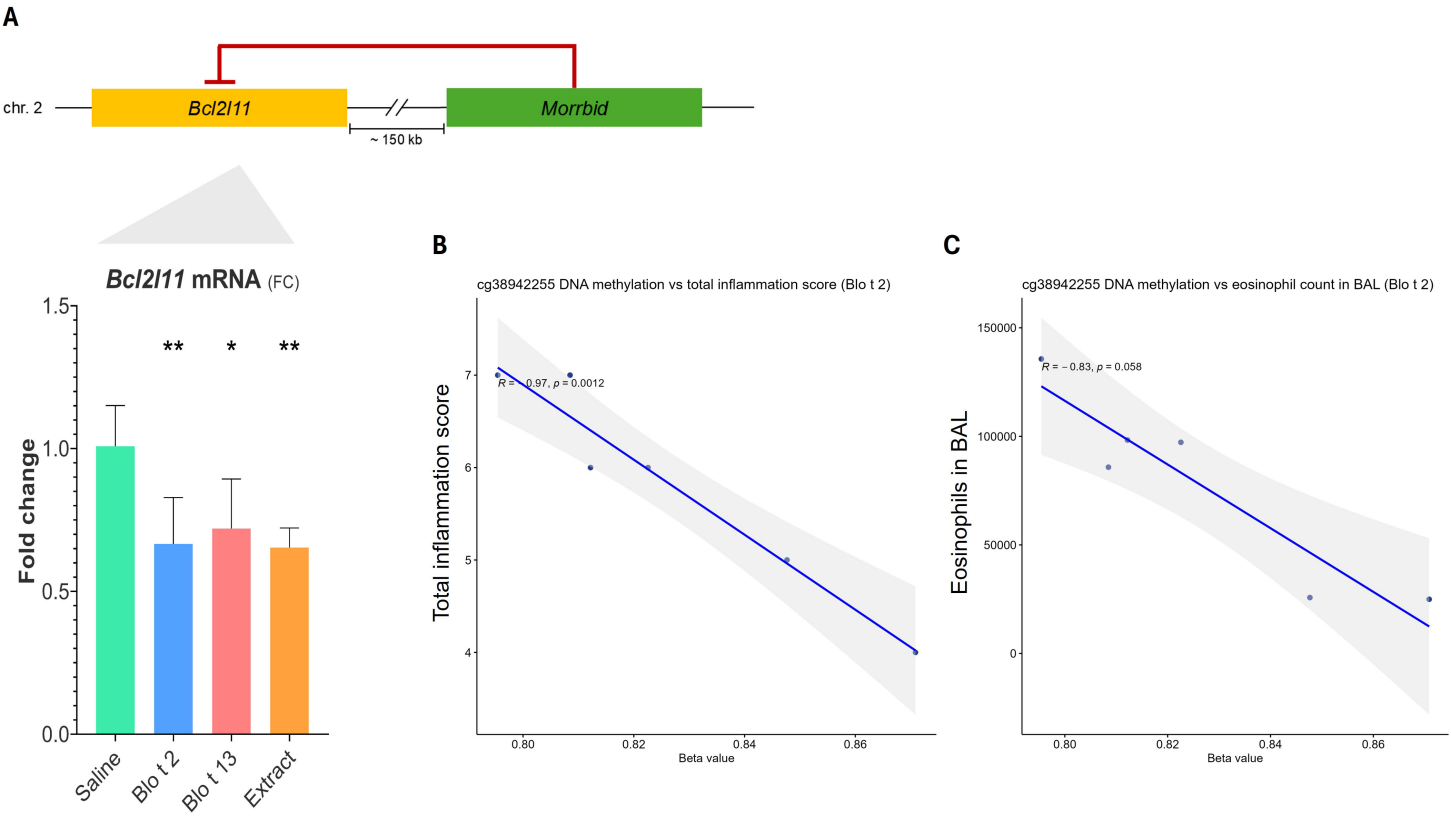
The Morrbid-Bcl2l11 axis is dysregulated upon allergen exposure. **(A)** A representative schema about the regulation of *Morrbid* and *Bcl2l11* at mice chromosome 2 and mRNA gene expression of *Bcl2l11* in lungs of saline- and Blo t 2-treated mice using β2 microglobulin as endogenous control. *p < 0.05; **p < 0.01; one-way ANOVA with Dunnet’s *post hoc* test of ΔCt values. **(B)** Scatter plot of the correlation between DNA methylation at the cg38942255 position and total inflammation score in Blo t 2-exposed mice. R; Spearman’s rank correlation coefficient. **(C)** Scatter plot of the correlation between DNA methylation at the cg38942255 position and eosinophil count in BAL in Blo t 2-exposed mice. R; Spearman’s rank correlation coefficient. Data are from six biologically independent mice per group.

We detected a significant DMR in chromosome 11 encompassing the gene encoding for the signal transducer and activator of transcription 5A (Stat5a), a molecule known to induce the expression of antiapoptotic genes of the Bcl2 family. There were also significant DMR in the *Bcl6* gene (**Table 3**). We found lower mRNA expression of the gene BCL2 like 11 (*Bcl2l11*) in the lung tissue of all allergen-exposed groups (**Figure 7A**). We also found that DNA methylation levels in a CpG site cg38942255 in the non-coding RNA *Morrbid* were inversely correlated with the total inflammation score and the eosinophil cell counts in BAL fluid (**Figures 7B, C**). These findings together with differential DNA methylation in other genes such as SHC adaptor protein 1 (*Shc1*) suggest that exposure to *B. tropicalis* allergens may influence DNA methylation genes in key molecules involved in lung eosinophilic inflammation.

## 4 Discussion

In this study, we report for the first time that exposure to *B. tropicalis* allergens is associated with changes in lung DNA methylation in an acute model of allergic sensitization and airway inflammation. We developed an experimental model in which allergen exposure resulted in bronchial hyperreactivity, leukocyte tissue infiltration, and the production of allergen-specific antibodies. The resulting dataset was representative of a phenotype-enriched subset of animals showing concordant allergic airway inflammation compared to saline-exposed controls. In this study, Blo t 2 exposure was more associated with eosinophil and neutrophil infiltration, while Blo t 13 (a fatty acid binding protein) induced a greater number of goblet cells and fewer neutrophil infiltrates. These differences may reflect the distinct allergenic capacities and biochemical properties of each allergen. When analyzing DNA methylation changes, we found that some were significant in all allergens tested (such as DMRs in protocadherin alpha gene), while other DMRs were specific to certain allergens.

Our study demonstrated significant DNA methylation differences in protocadherin alpha genes (*Pcdha1; Pcdha2; Pcdha3; Pcdha4; Pcdha5; Pcdha6*) upon allergen exposure. These changes were observed in all mice exposed to *Blomia tropicalis* extract and to the purified allergens; spanning about 26 CpG sites on mouse chromosome 18. The decrease in DNA methylation was more pronounced in mice treated with Blo t 13 and *B. tropicalis* extract. Our findings are biologically plausible, as protocadherin-1 was initially identified as a gene associated with bronchial hyperreactivity (49) and later confirmed as an asthma susceptibility gene in several studies (50–52). Although mainly expressed in neurons, protocadherins have also been detected in airway epithelium (53), where they contribute to barrier functions (54). Previous studies have reported transcript changes in protocadherin levels following HDM exposure (55), and protocadherins can be upregulated by prostaglandins and leukotrienes in the context of type 2 inflammation (56). Furthermore, protocadherin-1 interacts with SMAD3, a molecule involved in TGF-beta signaling, and this interaction may affect epithelial barrier function, a critical aspect of asthma pathophysiology (57). Our pathway analysis of differentially methylated loci also revealed significant enrichment of protocadherin genes in cell adhesion pathways. Alpha protocadherins play important roles in neuronal migration, differentiation, and synaptogenesis; changes in DNA methylation in the gene encoding protocadherin-20 has been previously reported in sputum samples of asthmatic patients (58). The results of the present study suggests that methylation changes in protocadherin genes may occur upon allergen exposure. When we assessed mRNA expression of *Pcdha1*, *Pcdha4* and *Pcdha6* in mice lung, we found that *Pcdha4* was detected in mouse lung tissue (data not shown).

In this model a DMR was found in chromosome 4 near the loci encoding *IL11ra*. There are several experimental and mechanistic studies linking IL-11 receptor alpha signaling to asthma-like phenotypes. IL-11 itself is up-regulated in human asthmatic airways and correlate with disease severity. IL-11 mRNA and protein are increased in epithelial and subepithelial cells and expression correlate with fibrosis and a decrease in forced expiratory volume. In mouse models, type 2 cytokines induce the IL-11R signaling pathway contributing to antigen induced sensitization, eosinophilic inflammation and airway mucus production (59). Other studies also reported that IL-11 signaling is involved in airway response to methacholine after acute exposure to ozone (60).

Beyond classical immune and structural signaling, our global functional annotation of genes harboring DMRs revealed significant enrichment in pathways related to nerve growth factor processing and neurotrophin expression and processing, suggesting a connection between allergen exposure and neuronal-related pathways. Our results are in agreement with other DNA methylation studies on asthma mouse model in which several of the genes mapping to DMRs and differentially expressed genes were enriched in pathways associated with neuronal signaling and sensory nerve mediated bronchoconstriction (61) and, with earlier studies showing that genes involved in neuronal wiring, neuroendocrine regulation and neuronal cAMP signaling are perturbed in asthma (62).

We analyzed mRNA levels in some candidate genes involved in neuronal related functions. *Gdf7* encodes a growth factor that functions as a secreted ligand of the TGF-beta (transforming growth factor-beta) superfamily of proteins and binds various TGF-beta receptors, leading to recruitment and activation of SMAD family transcription factors that regulate gene expression. Our results also replicate previous findings suggesting that TGF-beta related genes are epigenetically modified after HDM exposure (19). Indeed, *Gdf7* was found to be less expressed in allergen exposed mice. Along with *Gdf7*, other genes such as *Naalad2* and *Cbln1* have been involved in neuronal growth and neuroinflammation (63, 64). *Naalad2* is typically expressed in discrete brain regions and encodes an enzyme with dipeptidase activity (N-acetylated alpha-linked acidic dipeptidase 2) and has not previously been associated with airway allergic inflammation in a mouse model. In addition, a notable finding was the detectable mRNA expression of cerebellin 1 (*Cbln1*), a synaptic organizer belonging to the C1q family that promotes axon growth and serves as a guidance cue (65), only in animals exposed to Blo t 2. The expression of cerebellin 1 was initially reported as a glycoprotein secreted in the cerebellum, where it is required for proper synaptic function between parallel fibers and Purkinje cells, but it has also been found in other brain regions involved in axon guidance and synaptogenesis (66). Prune Homolog 2 (*Prune2*) is highly expressed in the nervous system and is a critical regulator of neuronal survival, apoptosis, and synapse maintenance. It helps prevent neurodegeneration and keeps established neuronal networks viable. Our results suggest that HDM exposure is associated with changes in the expression of these molecules in lung tissue, which may be relevant to the airway inflammatory process and neural innervation. Nevertheless, these neuron-associated transcripts were detected in whole lung tissue, reflecting the cellular heterogeneity of this organ and the contribution of minor cellular populations, their differential methylation and expression may still reflect biologically relevant regulatory changes in specific cell subsets.

We hypothesize that differentially methylated signals and genes expression differences may come from pulmonary neuroendocrine cells (PNECs), which reside in the lung epithelium and communicate directly with the brain via the vagus nerve, and locally with immune cells. The changes in synaptic organizers (*Cbln1*, *Cdh8*), survival genes (*Prune2*), and calcium sensors (*Ncald*) are likely not happening in the standard lung structural cells, but rather in the local airway nerve fibers and PNECs. The allergen exposure is likely rewiring the lung’s local nervous system, making it hyper-sensitive to triggers—a classic hallmark of asthma. Allergen exposure was also associated with significant changes in DNA methylation of immune related genes. PNECs and local nerves release neuropeptides that directly activate mast cells and T-cells, which in turn release leukotrienes and prostaglandins, thereby this epigenetic footprint shows that allergen exposure may involve both the “sensors” (the neural genes) and the “responders” (the immune genes), locking the lung into a state of chronic neurogenic inflammation.

Moreover, we found several DMR related with the Bcl family and the reduced expression of *Bcl2l11* in allergen sensitized mice (**Figure 7**). *Bcl2l11* encodes a proapoptotic protein named Bim that is critical to limit type 2 inflammation. The reduced expression of Bcl2l11 may prolong survival of pathogenic T cells and eosinophils in the airway. Previous studies have shown increased expression of Bcl-2 in eosinophils from sputum in patients with severe asthma that may prolong survival and decrease apoptosis of airway eosinophils (67). Recent studies have found association of genetic variants with asthma risk (68). In this study DNA methylation levels in the non-coding RNA *Morrbid* (which regulates *Bcl2l11*) were correlated with eosinophil cell counts in BAL. *Morrbid* is also present in humans and is dysregulated in patients with hypereosinophilic syndrome; this non-coding RNA tightly controls the survival of neutrophils, eosinophils and “classical” monocytes in response to pro-survival cytokines by regulating in cis the transcription of its neighboring pro-apoptotic gene *Bcl2l11* (Bim) (69).

**Figure 8:**
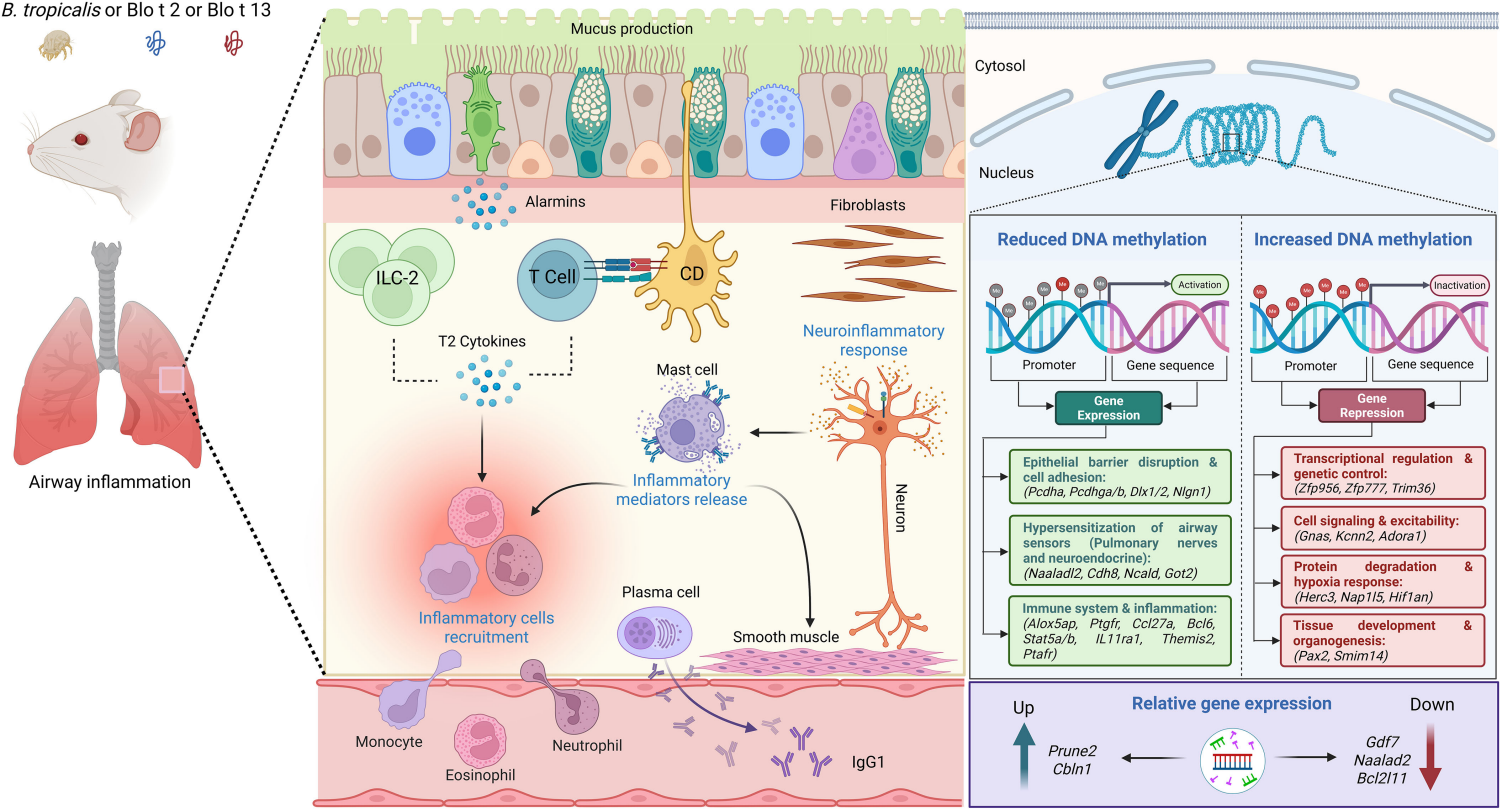
A summary on the cellular and epigenetic changes associated with exposure to *Blomia tropicalis* allergens in a murine model.

The aforementioned gene expression analyses were conducted as exploratory transcriptional follow-up of prioritized methylation signals, were not intended as independent confirmatory validation and should be interpreted as hypothesis-generating. Although multiple-testing correction was applied across all qPCR targets, the limited sample size warrants cautious interpretation and independent validation in larger cohorts will be required to confirm these associations. A summary illustrating the inflammatory readouts after exposure to *Blomia tropicalis* extract, Blo t 2, and Blo t 13 and the main genes and pathways detected by the DNA methylation and gene expression analysis are presented in **Figure 8**.

Nevertheless, the findings of this study have to be seen in light of some limitations. First, DNA methylation analyses were performed in a small subset of animals that were selected based on successful sensitization, which may introduce selection bias and restrict generalizability. Thereby, the reported associations should be interpreted as epigenetic changes occurring during an inflammatory process, in animals with robust allergic airway responses. Although this extreme phenotype or *responder-only* sample selection approach enhances biological signal detection in exploratory epigenetic analyses, it limits population-level causal inference and therefore, the reported associations are hypothesis-generating and warrant validation in larger cohorts. Second, we here analyzed bulk lung tissue methylation and differences in cell composition may influence the detected differences between allergen exposed mice and saline controls, moreover, we cannot differentiate if the observed methylation changes were occurring in the epithelium, in the stroma or in the infiltrating inflammatory cells. However, to partially account for unmeasured confounding, we included the first two principal components in our models, which capture major sources of variation and may mitigate the impact of cell-type differences and reported associations were corrected by genomic inflation.

In conclusion, exposure to *Blomia tropicalis* allergens is associated with changes in DNA methylation and mRNA expression in mouse lung, and the genes that were epigenetically modified encode several proteins involved in cell adhesion and neuroinflammation pointing to key genes of neuroimmune cross-talk alarm. Some DNA methylation changes observed after exposure to the complete extract of *Blomia tropicalis*, were also observed in mice sensitized with the purified allergens, however, there were also allergen-specific effects on the mouse methylome. Our data also show clear differences in DNA methylation between allergen-exposed and control groups of mice with the same genetic background. Future studies are needed to accurately define the cellular origin of these changes and their stability. Overall, our study provides new insights on the DNA methylation and mRNA expression changes in mice lung following exposure to *Blomia tropicalis* allergens, suggesting new genes and potential pathways underlying the acute response to house dust mite allergens and revealing key molecules in cell adhesion and neuroinflammation.

## Data Availability

The datasets presented in this study can be found in online repositories. The names of the repository/repositories and accession number(s) can be found in the article/**Supplementary Material**.
