# Supplementary material for: Blomia tropicalis allergens induce lung DNA methylation changes in neuroimmune genes in a mouse model of airway inflammation
Source: Front Immunol. 2026 Jul 2;17:1775662. doi: 10.3389/fimmu.2026.1775662 (PMC13372613; doi:10.3389/fimmu.2026.1775662)
Supplement: Supplementary file 2 [file DataSheet2.pdf]

````
---
title: Blomia tropicalis allergens induce lung DNA methylation changes in a mouse
  model of airway inflammation
output: html_document
date: "2026-03-19"
---

```{r setup, include=FALSE}
rm(list = ls());gc()
knitr::opts_chunk$set(echo = TRUE)
```

```{r library, include=FALSE}


library(sesame)
library(sesameData)
library(ggplot2)
library(ENmix)
library(ggbiplot)
library(IlluminaMouseMethylationanno.12.v1.mm10)
library(minfi)
library(dplyr)
library(readr)
library(readxl)
library(bacon)
```



##Pre-processing and QC
```{r Pre-processing of MM285 data}

############################################################
# 1. Annotation (Illumina Mouse mm10)
############################################################
setwd("P:/Pediatrik_Melén/Simon_folder/metBtmice/analysis/repositorio/repositorio")
annotation.t <- minfi::getAnnotation(IlluminaMouseMethylationanno.12.v1.mm10) %>%
  as.data.frame()

annotation.t <- annotation.t[, c("Name", "chr", "pos",
                                 "Relation_to_Island",
                                 "GeneName_NCBI",
                                 "Feature_NCBI")]

colnames(annotation.t) <- c("probes", "chr", "pos",
                           "Relation_to_Island",
                           "GeneName_NCBI",
                           "Feature_NCBI")


###meta data##
Metadata_BtMiceMethylation <- read_excel("Metadata_BtMiceMethylation.xlsx")
SampleAnnotation<- read_delim("1_SampleAnnotation.txt", delim = "\t", escape_double = FALSE, trim_ws = TRUE)
colnames(SampleAnnotation)<-c("ChIP_ID","Mouse_ID")
metadata<- SampleAnnotation %>% inner_join(Metadata_BtMiceMethylation,by="Mouse_ID")

# Original group
metadata$group <- metadata$Group

````
````

```
# Recode to desired labels
metadata$group[grepl("Saline", metadata$Group)] <- "Saline"
metadata$group[grepl("Blo t 2", metadata$Group)] <- "Blo t 2"
metadata$group[grepl("Blo t 13", metadata$Group)] <- "Blo t 13"
metadata$group[grepl("Blomia tropicalis extract", metadata$Group)] <- "Ext"

# Convert to factor (important for PCA / models)
metadata$group <- factor(metadata$group,
                              levels = c("Saline", "Blo t 2", "Blo t 13", "Ext"))


############################################################
# 1. Load manifest (mouse array)
############################################################

mft <- sesameDataGet("MM285.address")$ordering

############################################################
# 2. PCA BEFORE QC
############################################################

# Get minimally processed data (NO QC)
betas_raw <- openSesame(
  ".",
  prep = "",
  manifest = mft
)

# Convert to M-values
Mvals_raw <- B2M(betas_raw)

# Remove probes with NA
Mvals_raw <- Mvals_raw[complete.cases(Mvals_raw), ]

# Transpose: samples as rows
Mvals_raw_t <- t(Mvals_raw)



# Use clean group variable
groups <- metadata$group

# PCA
pca_raw <- prcomp(Mvals_raw_t, center = TRUE, scale. = TRUE)

# Plot BEFORE QC
p_before <- ggbiplot(pca_raw,
                     ellipse = TRUE,
                     groups = groups,
                     var.axes = FALSE) +
  ggtitle("PCA Before QC") +
  theme_classic() +
  theme(legend.position = "bottom")

print(p_before)

############################################################
# 3. PCA AFTER QC (TQCD0PB pipeline)
############################################################

# Apply QC + normalization
```

```
betas_qc <- openSesame(
  ".",
  prep = "TQCD0PB",
  manifest = mft
)

# Convert to M-values
Mvals_qc <- B2M(betas_qc)

# Remove NA probes
Mvals_qc <- Mvals_qc[complete.cases(Mvals_qc), ]

# Transpose
Mvals_qc_t <- t(Mvals_qc)


# PCA
pca_qc <- prcomp(Mvals_qc_t, center = TRUE, scale. = TRUE)

# Plot AFTER QC
p_after <- ggbiplot(pca_qc,
                    ellipse = TRUE,
                    groups = groups,
                    var.axes = FALSE) +
  ggtitle("PCA After QC (TQCD0PB)") +
  theme_classic() +
  theme(legend.position = "bottom")

print(p_after)

############################################################
# 4. Save plots
############################################################

#ggsave("PCA_before_QC.png", plot = p_before,
#       width = 8, height = 6, dpi = 300)

#ggsave("PCA_after_QC.png", plot = p_after,
 #      width = 8, height = 6, dpi = 300)


## we use screen plot to determine the number of PC, we select the first 2 PC,PC1 and PC2 after the QC using the elbow rule.

# Create dataframe
scree_df <- data.frame(
    PC = paste0("PC", 1:length(pca_qc$sdev)),
    Variance = (pca_qc$sdev^2 / sum(pca_qc$sdev^2)) * 100
)

# Correct ordering of PCs
scree_df$PC <- factor(scree_df$PC, levels = paste0("PC", 1:length(pca_qc$sdev)))

# Line plot for scree
ggplot(scree_df[1:10, ], aes(x = PC, y = Variance, group = 1)) +
    geom_line(color = "steelblue", size = 1) +
    geom_point(color = "red", size = 3) +
    geom_text(aes(label = round(Variance, 1)), vjust = -0.5, size = 3) +
    theme_classic() +
    ylab("Variance Explained (%)") +
    xlab("Principal Component") +
```

````
    ggtitle("Scree Plot (After QC)") +
    theme(axis.text.x = element_text(angle = 45, hjust = 1))

##add the PC in metaData

metadata<-cbind(metadata,pca_qc$x[,c("PC1","PC2")])

```

##Pre-processing and QC each step to remove CpGs for  "TQCD0PB"
```{r Pre-processing each step of MM285 data}

library(sesame)

# Load raw data
sdf_list <- openSesame(".", prep = "", manifest = mft, func = NULL)

# Apply steps one by one for  TQCD0PB
sdf0 <- sdf_list
sdf1 <- lapply(sdf0, inferStrain)
sdf2 <- lapply(sdf1, qualityMask)
sdf3 <- lapply(sdf2, inferInfiniumIChannel)
sdf4 <- lapply(sdf3, dyeBiasNL)
sdf5 <- lapply(sdf4, resetMask)
sdf6 <- lapply(sdf5, pOOBAH)
sdf7 <- lapply(sdf6, noob)

count_masked <- function(sdf_list) {
    sapply(sdf_list, function(sdf) sum(sdf$mask, na.rm = TRUE))
}

mask_counts <- data.frame(
    raw = count_masked(sdf0),
    inferStrain = count_masked(sdf1),
    qualityMask = count_masked(sdf2),
    inferInfiniumI = count_masked(sdf3),
    dyeBias = count_masked(sdf4),
    resetMask = count_masked(sdf5),
    pOOBAH = count_masked(sdf6),
    noob = count_masked(sdf7)
)

print(mask_counts)

count_mask_stats <- function(sdf_list) {
    sapply(sdf_list, function(sdf) {
        total <- nrow(sdf)
        masked <- sum(sdf$mask, na.rm = TRUE)
        pct <- round(masked / total * 100, 2)
        c(total = total, masked = masked, percent = pct)
    })
}

mask_stats <- as.data.frame(t(count_mask_stats(sdf6)))
print(mask_stats)


write.table(mask_counts,
            file = "Pre.processQC.flaged.CpGs.txt",
            sep = "\t",
            row.names = FALSE,
````

````
            quote = FALSE)

write.table(mask_stats,
            file = "Pre.processQC.masked.CpGs.txt",
            sep = "\t",
            row.names = FALSE,
            quote = FALSE)

```



## EWAS ANALYSES
```{r EWAS, echo=FALSE}


###################################################
#                  EWAS ANALYSES                  #
###################################################

library(MASS)
library(lmtest)
library(sandwich)
library(parallel)
library(dplyr)
meth.main<-Mvals_qc_t
# Ensure allergen factor has correct reference
metadata$group <- relevel(metadata$group , ref = "Saline")

###################################################
# FUNCTION: Run robust linear regression per CpG
###################################################
RLMtest <- function(meth_matrix , methcol, exposure, X1, X2) {
  mod <- try(rlm(meth_matrix[, methcol] ~ exposure + X1 + X2 , maxit = 200))
  cf  <- try(coeftest(mod, vcov = vcovHC(mod, type = "HC0")))

  if (inherits(mod, "try-error") | inherits(cf, "try-error")) {
    message(paste("Error in column", methcol))
    out <- data.frame(matrix(NA, nrow = 3, ncol = 3))
    colnames(out) <- c("Estimate", "Std.Error", "Pr(>|z|)")
  } else {
    out <- cf[2:4, c("Estimate", "Std. Error", "Pr(>|z|)")]
     # FIX: replace zero p-values
    pvals <- out[, "Pr(>|z|)"]
    pvals[pvals == 0] <- .Machine$double.xmin

    out[, "Pr(>|z|)"] <- pvals
  }

  return(out)
}

###################################################
# RUN EWAS using Windows-compatible parallelization
###################################################

# Number of cores
ncores <- detectCores() - 1
cl <- makeCluster(ncores)          # create cluster
clusterExport(cl, varlist = c("meth.main", "metadata", "RLMtest"))
clusterEvalQ(cl, {library(MASS); library(lmtest); library(sandwich)})
````

```
# Run parallel EWAS
system.time({
  ind.res <- parLapply(
    cl,
    setNames(seq_len(ncol(meth.main)), colnames(meth.main)),
    function(col) RLMtest(
      meth_matrix = meth.main,
      methcol = col,
      exposure = metadata$group,
      X1 = metadata$PC1,
      X2 = metadata$PC2
    )
  )
})

stopCluster(cl)  # Stop cluster when done

###################################################
# FUNCTION: Convert list of results to data frame
###################################################
process_indres <- function(indres_list, index, probe_names) {

  df <- data.frame(do.call(rbind, lapply(indres_list, function(x) x[index, ])))

  colnames(df) <- c("Estimate", "SD", "P_VAL")
  df$probeid <- probe_names

  # Remove NA
  df <- df[complete.cases(df), ]

  return(df)
}

# Extract results for each allergen
probe_names <- colnames(meth.main)

ind.res.Blot13_c1 <- process_indres(ind.res, 1, probe_names)
ind.res.Blot13_c1<-ind.res.Blot13_c1[,c("probeid","Estimate","SD","P_VAL")]
ind.res.Blot2_c1  <- process_indres(ind.res, 2, probe_names)
ind.res.Blot2_c1<-ind.res.Blot2_c1[,c("probeid","Estimate","SD","P_VAL")]
ind.res.Extract_c1 <- process_indres(ind.res, 3, probe_names)
ind.res.Extract_c1<-ind.res.Extract_c1[,c("probeid","Estimate","SD","P_VAL")]
# Save Blot13
write.table(ind.res.Blot13_c1,
            file = "EWAS_Blot13_results.txt",
            sep = "\t",
            row.names = FALSE,
            quote = FALSE)

# Save Blot2
write.table(ind.res.Blot2_c1,
            file = "EWAS_Blot2_results.txt",
            sep = "\t",
            row.names = FALSE,
            quote = FALSE)

# Save Extract
write.table(ind.res.Extract_c1,
            file = "EWAS_Extract_results.txt",
            sep = "\t",
```

````
      row.names = FALSE,
      quote = FALSE)

```



### Genomic inflation correction using BECON

```{r BECON, echo=FALSE}

####################################################
# DNA methylation EWAS - BACON correction
####################################################

library(bacon)
library(qqman)

####################################################
# FUNCTION: Adjust p-values + CI + filtering
####################################################
adjust_pvals <- function(df, method = "BH") {

  # Fix p-values = 0
  df$P.Value[df$P.Value == 0] <- .Machine$double.xmin

  # FDR correction
  df$FDR_BH <- p.adjust(df$P.Value, method = method)

  # Add 95% CI
  df$CI_lower <- df$Estimate - 1.96 * df$SD
  df$CI_upper <- df$Estimate + 1.96 * df$SD

  # Significant CpGs
  df$Significant.CpGs <- ifelse(abs(df$Estimate) >= 1.4 & df$FDR_BH < 0.05, "Yes", "No")

  return(df)
}

####################################################
# FUNCTION: Inflation factor
####################################################
inflation_p <- function(ps) {
  ps[ps == 0] <- .Machine$double.xmin
  chisq <- qchisq(1 - ps, 1)
  lambda <- median(chisq) / qchisq(0.5, 1)
  return(lambda)
}

####################################################
# MAIN LOOP
####################################################

for (i in c("EWAS_Blot13_results", "EWAS_Blot2_results", "EWAS_Extract_results")) {

  cat("Processing:", i, "\n")

  ####################################################
  # Load data (FIXED sep)
  ####################################################
````

```

  Data_result <- read.table(
    paste0(i, ".txt"),
    header = TRUE,
    sep = "\t",
    stringsAsFactors = FALSE
  )

  ###################################################
  # Extract values
  ###################################################

  BETA   <- Data_result$Estimate
  SE     <- Data_result$SD
  probes <- Data_result$probeid

  ###################################################
  # BACON correction
  ###################################################

  bc <- bacon(NULL, BETA, SE)

  tstat <- tstat(bc)
  pval  <- pval(bc)
  coef  <- es(bc)
  se    <- se(bc)

  ###################################################
  # Create dataframe
  ###################################################

  becon.df <- data.frame(
    probes = probes,
    Estimate = coef,
    SD = se,
    t = tstat,
    P.Value = pval
  )

  ###################################################
  # Adjust p-values + CI
  ###################################################

  res <- adjust_pvals(becon.df, method = "BH")

    ###################################################
  # Add annotation from illumina for mouse using mm10
  ###################################################
  # Ensure no duplicate probes in annotation
  annotation.t <- annotation.t %>% distinct(probes, .keep_all = TRUE)

 # Join
  res <- annotation.t %>% right_join(res, by = "probes")


  ###################################################
  # Save full results
  ###################################################

  write.table(res,
              file = paste0(i, "_bacon.txt"),
```

```
                col.names = TRUE,
                row.names = FALSE,
                sep = "\t",
                quote = FALSE)

    ####################################################
    # QQ plot + lambda
    ####################################################

  # Function to create QQ plot
  plot_qq <- function(pvals, title = "QQ plot") {

    # Fix p-values = 0
    pvals[pvals == 0] <- .Machine$double.xmin

    # Expected vs observed
    observed <- -log10(sort(pvals))
    expected <- -log10(ppoints(length(pvals)))

    qq_df <- data.frame(expected = expected, observed = observed)

    # Lambda
    lambda <- inflation_p(pvals)

    # Plot
    p <- ggplot(qq_df, aes(x = expected, y = observed)) +
      geom_point(size = 1.5, alpha = 0.6) +
      geom_abline(intercept = 0, slope = 1, color = "red") +
      theme_classic() +
      ggtitle(title) +
      xlab("Expected -log10(p)") +
      ylab("Observed -log10(p)") +

      # Add lambda text
      annotate("text",
               x = max(expected)*0.6,
               y = max(observed)*0.9,
               label = paste0("lambda = ", signif(lambda, 3)),
               size = 5)

    return(p)
  }

  p <- plot_qq(becon.df$P.Value, title = paste0("QQ plot: ", i))

  ggsave(filename = paste0("QQ_", i, "_bacon.png"),
         plot = p,
         width = 6,
         height = 6,
         dpi = 300)

    ####################################################
    # Inflation comparison
    ####################################################

    cat("Inflation BEFORE correction:",
        inflation_p(Data_result$P_VAL), "\n")

    cat("Inflation AFTER correction:",
        inflation_p(res$P.Value), "\n\n")
  }
```

````


#Processing: EWAS_Blot13_results
#Inflation BEFORE correction: 1.574923
#Inflation AFTER correction: 1.23087

#Processing: EWAS_Blot2_results
#Inflation BEFORE correction: 1.366553
#Inflation AFTER correction: 1.172903

#Processing: EWAS_Extract_results
#Inflation BEFORE correction: 1.731584
#Inflation AFTER correction: 1.248494


```


## DMR analysis using DMRcate

```{r DMR, echo=FALSE}


library(dplyr)
library(IlluminaMouseMethylationanno.12.v1.mm10)
annotation.table <- minfi::getAnnotation(IlluminaMouseMethylationanno.12.v1.mm10)%>%as.data.frame()
annotation.table<-annotation.table[,c("Name","chr","pos")]
colnames(annotation.table)<-c("probes","chr","start")
annotation.table$end<-annotation.table$start+1
library(readr)
library(GenomicRanges)
library(stats4)
library(BiocGenerics)
library(parallel)
library(S4Vectors)
library(rGREAT)
library(tidyverse)
set.seed(123)

for (f in c("EWAS_Blot13_results", "EWAS_Blot2_results", "EWAS_Extract_results")) {

  indiv.results <- read_delim(paste0(f,"_bacon.txt"),
                              delim = "\t", escape_double = FALSE, trim_ws = TRUE) ##read your meta-analysis file
  ## your file contains probID:(CpGID) chr (chromosome number) pos (CpGs position) BETA (coefficient) SE (standard error) P_VAL (p-value)


  inputfileBed <- indiv.results[,c("probes","Estimate","SD","P.Value")]
  rownames(inputfileBed)<-inputfileBed$probes

  inputfileBed<-left_join(inputfileBed, annotation.table, by = "probes")
  inputfileBed<-inputfileBed[c("probes","Estimate","SD","P.Value","chr","start","end" )]
  colnames(inputfileBed)<-c("probID","BETA","SE","P_VAL","chr","start","end")

  # Keep only autosomal chromosomes (1-19 for mouse)
  inputfileBed <- inputfileBed[which(inputfileBed$chr %in% paste0("chr", 1:19)),]

````

```
#inputfileBed$pos<-as.integer(inputfileBed$pos) ## set the position as integer
inputfileBed$stat<-inputfileBed$BETA/inputfileBed$SE ## based on coef and se we creat t stattistics
inputfileBed$probID<-as.character(inputfileBed$probID)## set CpGs ID to character
#inputfileBed$chr<-paste("chr",as.character(inputfileBed$chr),sep="") ## add "chr" to the chromosome number
inputfileBed$indfdr<-p.adjust(inputfileBed$P_VAL, "BH") ## calculate fdr on the p-value
inputfileBed$is.sig<-"FALSE"
inputfileBed$is.sig<-replace(inputfileBed$is.sig,inputfileBed$indfdr<0.05,"TRUE")
inputfileBed<-inputfileBed[,c("probID","stat","chr","start","end","BETA","indfdr","is.sig")]
inputfileBed<-inputfileBed[complete.cases(inputfileBed),]
inputfileBed<-inputfileBed[order(inputfileBed$chr,inputfileBed$start),]
colnames(inputfileBed)<-c("ID","stat","CHR","start","end","betafc","indfdr","is.sig")

#Input parameters used in the DMRcate algorithm:

#Parameter        Value   Description
#lambda              1000            Gaussian kernel bandwidth for smoothed-function estimation.
#Gaps ≥ lambda between significant CpG sites will be in separate  DMRs.
#C            2      Scaling factor for bandwidth. Gaussian kernel is calculated
#where lambda/C = sigma. Empirical testing shows that, for 450k data when lambda = 1000, near-optimal prediction of sequencing-derived DMRs is obtained when C is approximately 2.
#Pcutoff               0.05       p-value cutoff to determine DMRs.
#min.cpgs               5         Minimum number of consecutive CpGs constituting a DMR.

library(DMRcate)
library(gtools)

annotated<-GRanges(as.character(inputfileBed$CHR), IRanges(inputfileBed$start, inputfileBed$end), stat = inputfileBed$stat, diff = inputfileBed$betafc, ind.fdr = inputfileBed$indfdr, is.sig = inputfileBed$indfdr < 0.05)
names(annotated) <-  rownames(inputfileBed)
datainput<-new("CpGannotated", ranges=annotated)
dmrcoutput <- dmrcate(datainput, lambda=1000, C=2,pcutoff=0.05,min.cpgs=5)
results.ranges <- extractRanges(dmrcoutput, genome = "mm10")
results.ranges_out<-data.frame(results.ranges)
results.ranges_out<-results.ranges_out[mixedorder(results.ranges_out$min_smoothed_fdr),]
print("Total number of significant DMR")
print(dim(results.ranges_out)[1])

library (plyr)
combp_cpg<-list()
for (i in 1:dim(results.ranges_out)[1]){
  combp_cpg[[i]]<-inputfileBed[which((inputfileBed$CHR==results.ranges_out$seqnames[i]) & (inputfileBed$start>=results.ranges_out$start[i]) &( inputfileBed$start<=results.ranges_out$end[i])),]

}
DMR_both_combp_cpg <- ldply (combp_cpg, data.frame)

```

```
    indiv.results.list<-list()
    for( j in 1:dim(results.ranges_out)[1]){
      data<-combp_cpg[[j]]
      CHR<- unique(data$CHR)
      start<-min(data$start)
      end<-max(data$end)
      ID<-paste(paste(CHR,start,sep=":"),end,sep="-")
      PROBE<-paste(as.vector(data$ID),collapse=",")
      N_PROBE<-length(data$ID)
      indiv.results.list[[j]]<-data.frame(chromosome_position=ID,N_CpGs=N_PROBE,CpGs_list=PROBE)
    }

    indiv.results.par <- ldply(indiv.results.list, rbind)
    indiv.results.par<- cbind(indiv.results.par,results.ranges_out)
    indiv.results.par<-indiv.results.par[,c("chromosome_position","N_CpGs","min_smoothed_fdr","meandiff","overlapping.genes","CpGs_list")]
    head(indiv.results.par)


    ## annotation of missing gene names in the DMR using rGREAT
    split_result <- do.call(rbind, strsplit(indiv.results.par$chromosome_position, "[:-]"))

    # Assign column names
    colnames(split_result) <- c("chr", "start", "end")
    # Convert to a data frame
    bed <- as.data.frame(split_result, stringsAsFactors = FALSE)

    # Optionally convert start and end to numeric
    bed$start <- as.numeric(bed$start)
    bed$end <- as.numeric(bed$end)
    head(bed)
    indiv.results.par.ann <-cbind(indiv.results.par, bed)



    ### get gene names using the position in the genomes 250kb up and down
    ##bed  ####data frame containes chr    start      end
    # Submit job with 250 kb upstream and downstream extension
    job <- submitGreatJob(
      bed,
      species = "mm10",
      rule = "basalPlusExt",
      adv_upstream = 0,
      adv_downstream = 0,
      adv_span = 250000  # 250 kb extension
    )
    plotRegionGeneAssociationGraphs(job)
    res <- getRegionGeneAssociations(job)
    res<-data.frame(res)

    great<-list()
    for (r in 1:nrow(bed)){
      s<-bed$start[r]
      e<- bed$end[r]
      great[[r]]<-data.frame(chr=as.character(res[which(res$start==s & res$end==e),"seqnames"][1]),start=s,end=e,gene_GREAT=paste(res[which(res$start==s &
```

````
    res$end==e),"annotated_genes"],collapse=","),dist_to_TSS=paste(res[which(res$start==s & res$end==e),"dist_to_TSS"],collapse=","))
  }
  great<-do.call(rbind.data.frame, great)

  # Merge the data frames based on chr, start, and end columns
  indiv.results.par.ann <-left_join(indiv.results.par.ann, great, by = c("chr", "start", "end"))

  ###Save the data###
  file<-paste0(f,"becon_DMR.txt")###  file path to save output

  write.table(indiv.results.par.ann, file =file,col.names = TRUE, row.names = FALSE,sep = "\t",append=FALSE,quote =FALSE)
}







```
````
````
