# Supplementary material for: Blomia tropicalis allergens induce lung DNA methylation changes in neuroimmune genes in a mouse model of airway inflammation
Source: Front Immunol. 2026 Jul 2;17:1775662. doi: 10.3389/fimmu.2026.1775662 (PMC13372613; doi:10.3389/fimmu.2026.1775662)
Supplement: Supplementary file 3 [file DataSheet3.docx]

Supplementary Material


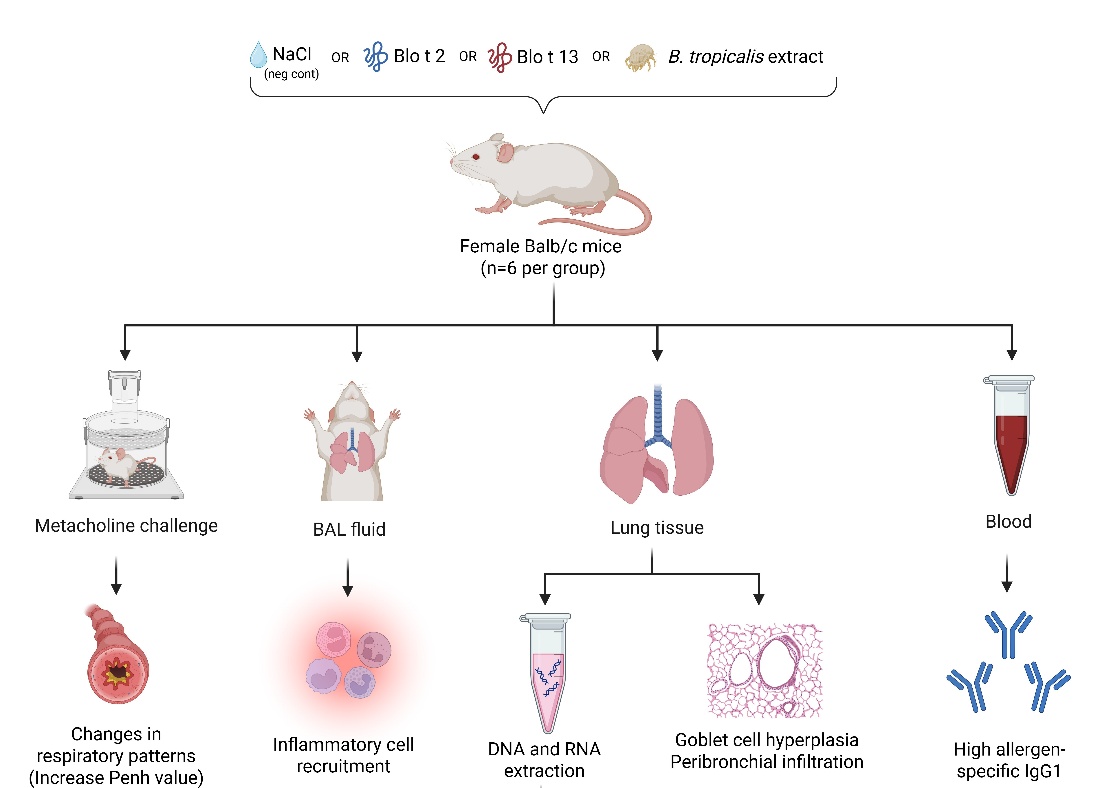


**Supplementary Figure S1.** Experimental procedures and samples collected from the allergen exposed mice. DNA was extracted from lung samples of 24 mice in which successful induction of acute airway inflammation was verified. Female BALB/c mice (n=6 per group) were exposed to either a negative control (NaCl), recombinant allergens (Blo t 2 or Blo t 13), or crude B. tropicalis extract. Following the sensitization and challenge phases, downstream analyses were conducted across four main physiological and molecular compartments: airway hyperresponsiveness was assessed via methacholine challenge, quantifying changes in respiratory patterns (manifested as an increase in Penh values). Bronchoalveolar lavage (BAL) fluid was collected to evaluate the recruitment of inflammatory cells into the airway spaces. Intact lung tissue was harvested and divided for histological examination (to assess peribronchial inflammatory infiltration and goblet cell hyperplasia) and for DNA/RNA extraction (to investigate underlying epigenetic and transcriptomic modifications). Blood serum was collected to measure the production of allergen-specific IgG1 antibodies, confirming a Th2-mediated systemic allergic response. Figure created in Biorender.


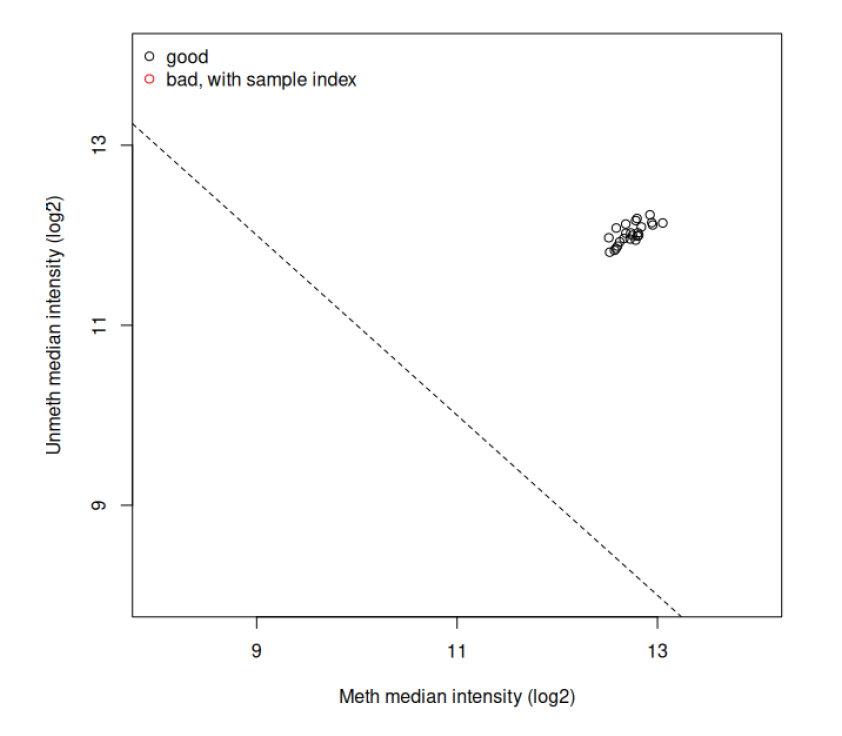


**Supplementary Figure S2.** Quality control plot of the log2 median intensity in the methylated (Meth) and unmethylated (Unmeth) channels for each sample of this study. The 24 samples analyzed were found to be of high-quality and clustered together in the upper right quadrant.


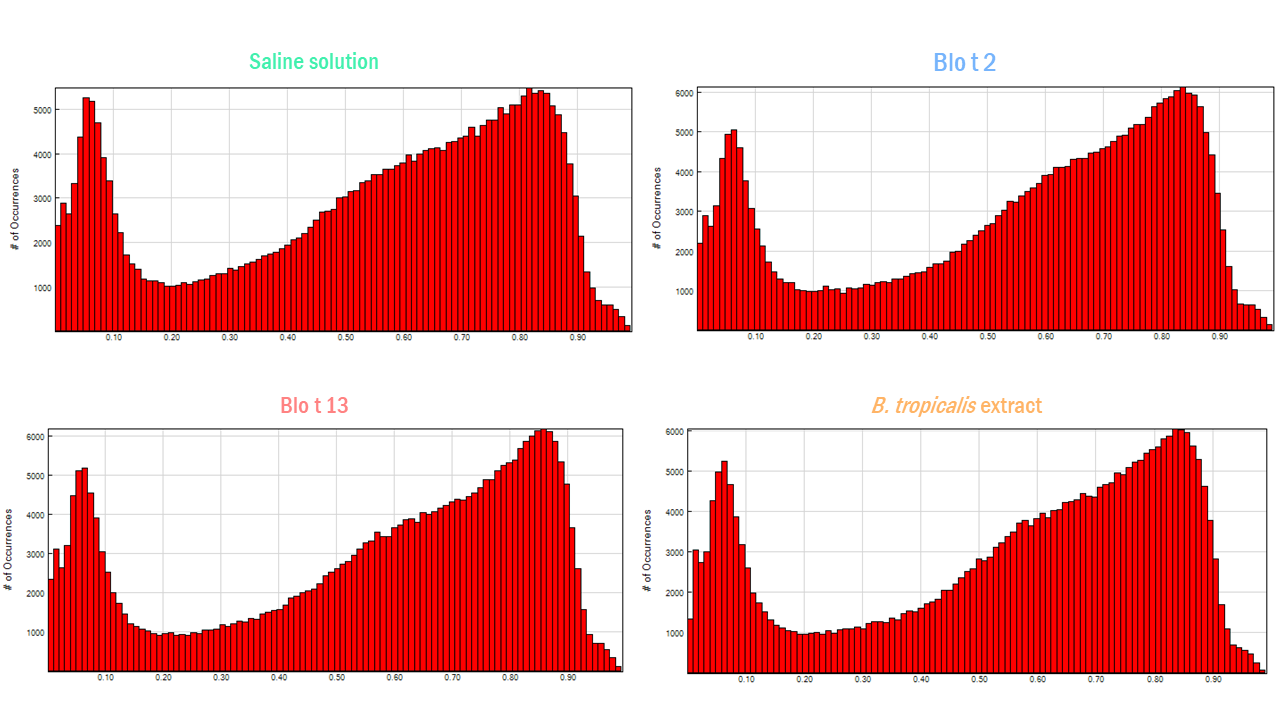


**Supplementary Figure S3.** Representative histograms of beta values in each group.


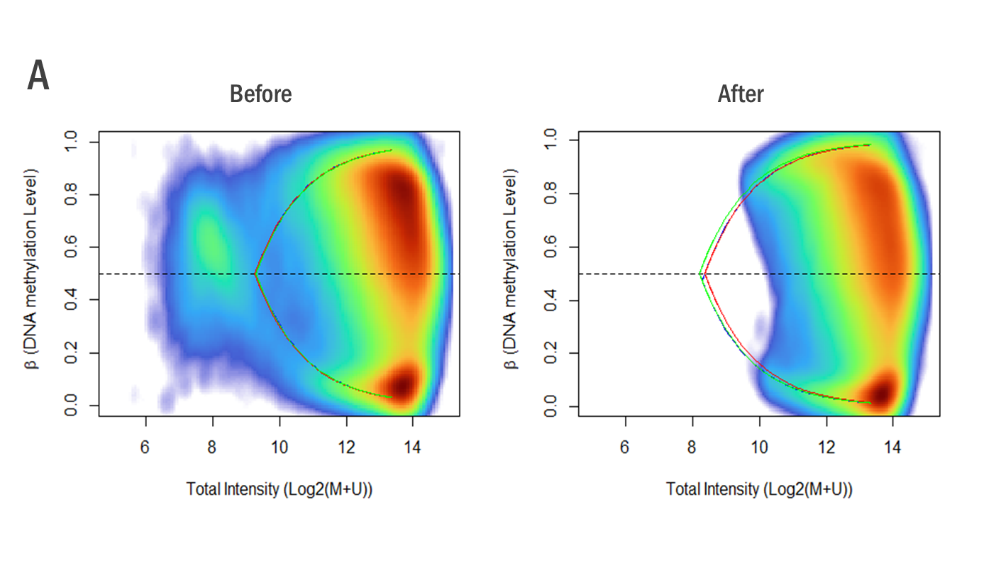


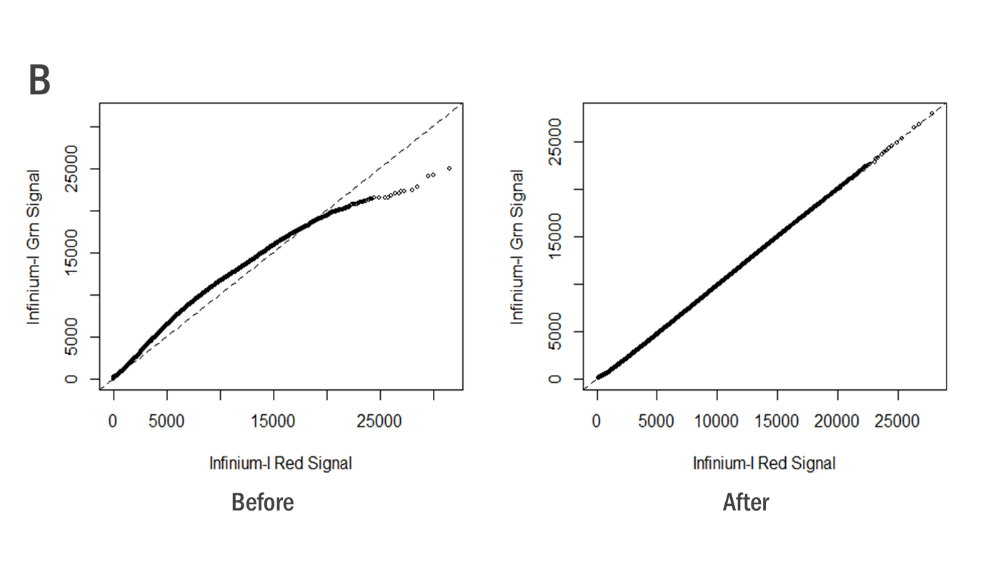


**Supplementary Figure S4.** Comparison of quality control plots before vs. after preprocessing. Representative graphs of the effects of preprocessing in quality control plots of a given sample. (**A**) Intensity vs. betas plots. (**B**) Red-Green quantile-quantile plots.


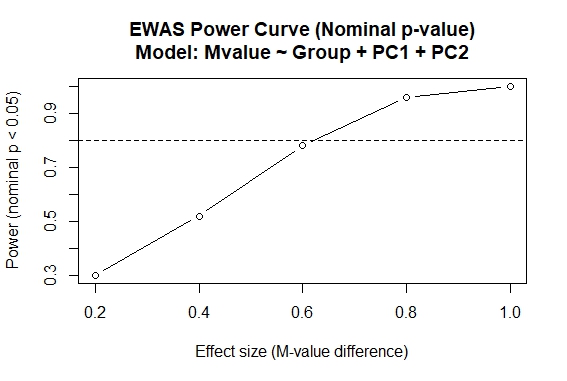


**Supplementary Figure S5.** Power calculation for M-value difference. We conducted a detailed simulation-based statistical power analysis for our EWAS design using the model: Mvalue ~ Group + PC1 + PC2 with four allergen groups. Power was evaluated using a nominal significance threshold (p < 0.05) for a range of M-value effect sizes, and all other CpGs were simulated as noise. These simulations indicate that large methylation differences are likely detectable, whereas smaller differences are underpowered at the given sample size.

1. **B.**


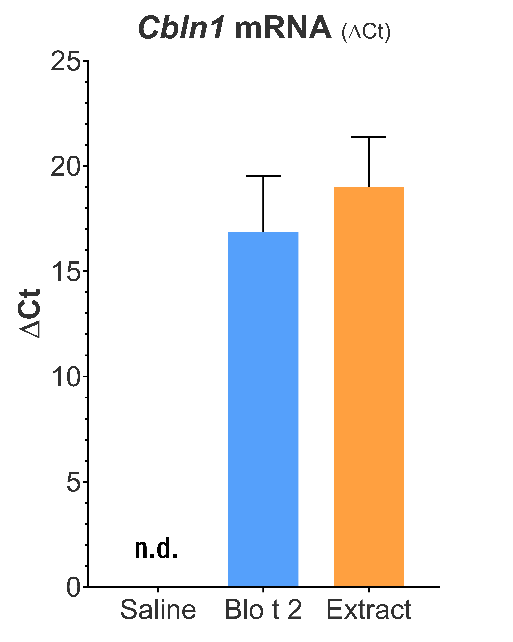

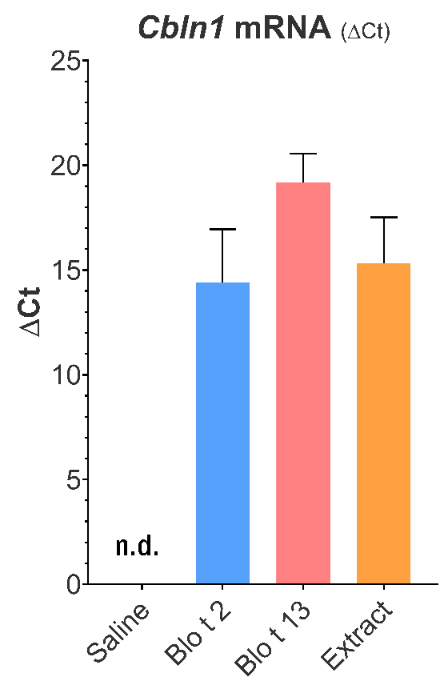


**Supplementary Figure S6**. mRNA gene expression of Cbln1 in all groups. Note that mRNA expression here is expressed as ΔCt since fold change values could not be calculated. A) cDNA synthesis with random hexamers. B) cDNA synthesis with oligo_dT_ primers.


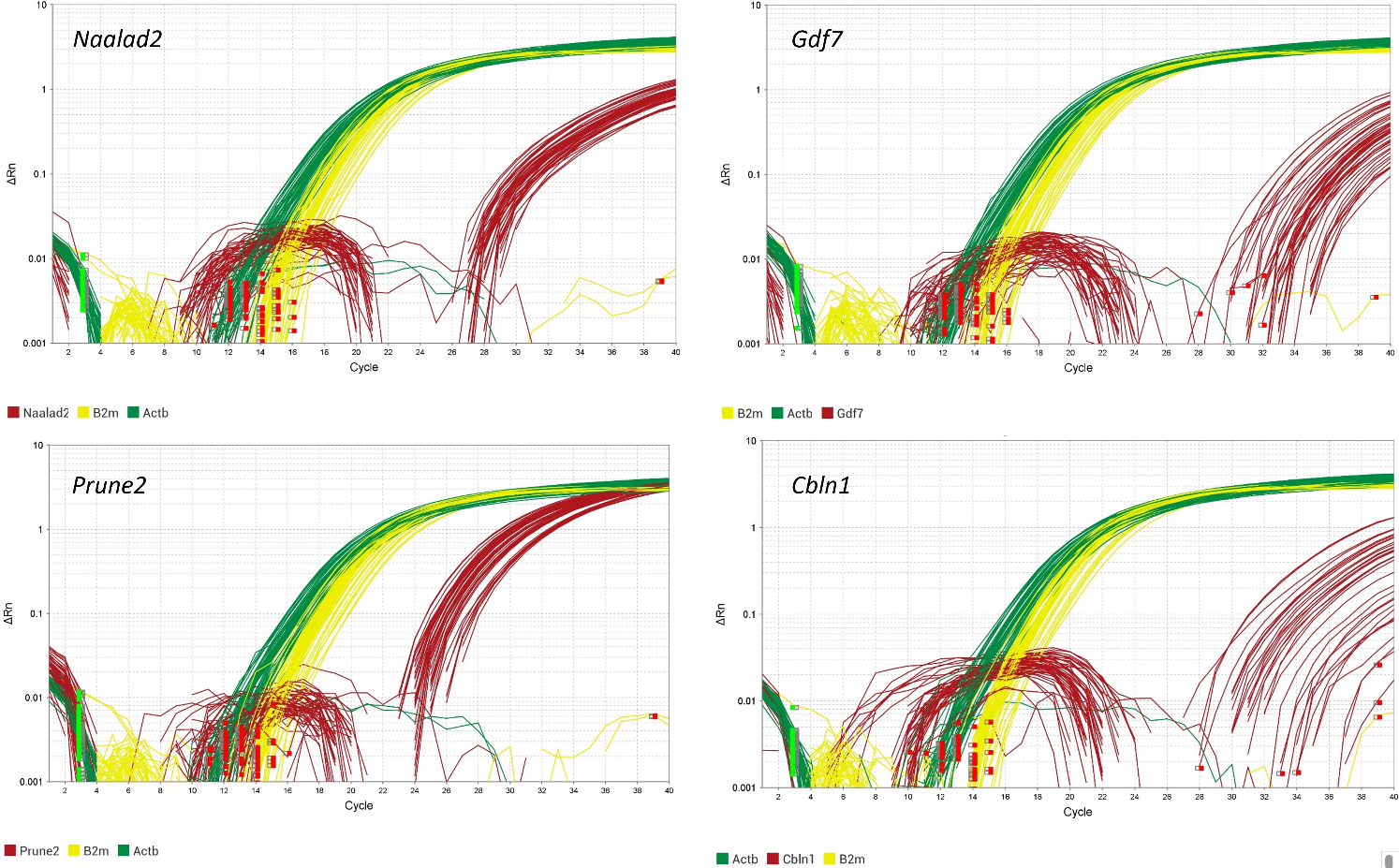


**Supplementary Figure S7**. Amplification plot for mRNA gene expression of 4 genes with significant differences in allergen-exposed mice and saline-controls. *Naalad2*: N-Acetylated alpha-linked acidic dipeptidase Like 2; *Gdf7*: Growth/Differentiation Factor 7; *Prune2*: Prune Homolog 2 with BCH Domain. *Cbln1*: Cerebellin 1 precursor. Measurements of mRNA expression of endogenous genes (*B2m* and *Actb*) are presented in yellow and green lines respectively, target genes are shown in red.

**Tables**

**Table S1. A QC flow table showing the number of probes removed or masked at each step of the TQCD0PB pipeline.**

| raw | inferStrain | qualityMask | inferInfiniumI | dyeBias | resetMask | pOOBAH | noob | total | masked | percent |
| --- | --- | --- | --- | --- | --- | --- | --- | --- | --- | --- |
| 11 | 14454 | 16065 | 16065 | 16065 | 0 | 10616 | 10616 | 296070 | 10616 | 3.59 |
| 12 | 14459 | 16069 | 16069 | 16069 | 0 | 10112 | 10112 | 296070 | 10112 | 3.42 |
| 23 | 14469 | 16080 | 16080 | 16080 | 0 | 10600 | 10600 | 296070 | 10600 | 3.58 |
| 15 | 14462 | 16074 | 16074 | 16074 | 0 | 10469 | 10469 | 296070 | 10469 | 3.54 |
| 17 | 14459 | 16071 | 16071 | 16071 | 0 | 10180 | 10180 | 296070 | 10180 | 3.44 |
| 30 | 14470 | 16082 | 16082 | 16082 | 0 | 9760 | 9760 | 296070 | 9760 | 3.3 |
| 19 | 14460 | 16071 | 16071 | 16071 | 0 | 9797 | 9797 | 296070 | 9797 | 3.31 |
| 47 | 14485 | 16095 | 16095 | 16095 | 0 | 9551 | 9551 | 296070 | 9551 | 3.23 |
| 16 | 14460 | 16072 | 16072 | 16072 | 0 | 10000 | 10000 | 296070 | 10000 | 3.38 |
| 19 | 14462 | 16074 | 16074 | 16074 | 0 | 9517 | 9517 | 296070 | 9517 | 3.21 |
| 5 | 14451 | 16063 | 16063 | 16063 | 0 | 11113 | 11113 | 296070 | 11113 | 3.75 |
| 8 | 14454 | 16065 | 16065 | 16065 | 0 | 9849 | 9849 | 296070 | 9849 | 3.33 |
| 0 | 14447 | 16059 | 16059 | 16059 | 0 | 9822 | 9822 | 296070 | 9822 | 3.32 |
| 0 | 14447 | 16059 | 16059 | 16059 | 0 | 9704 | 9704 | 296070 | 9704 | 3.28 |
| 10 | 14454 | 16066 | 16066 | 16066 | 0 | 9887 | 9887 | 296070 | 9887 | 3.34 |
| 7 | 14454 | 16065 | 16065 | 16065 | 0 | 9657 | 9657 | 296070 | 9657 | 3.26 |
| 26 | 14469 | 16080 | 16080 | 16080 | 0 | 9833 | 9833 | 296070 | 9833 | 3.32 |
| 17 | 14461 | 16073 | 16073 | 16073 | 0 | 9797 | 9797 | 296070 | 9797 | 3.31 |
| 20 | 14467 | 16079 | 16079 | 16079 | 0 | 10579 | 10579 | 296070 | 10579 | 3.57 |
| 10 | 14455 | 16066 | 16066 | 16066 | 0 | 9501 | 9501 | 296070 | 9501 | 3.21 |
| 19 | 14464 | 16074 | 16074 | 16074 | 0 | 10473 | 10473 | 296070 | 10473 | 3.54 |
| 10 | 14454 | 16066 | 16066 | 16066 | 0 | 9584 | 9584 | 296070 | 9584 | 3.24 |
| 5 | 14450 | 16062 | 16062 | 16062 | 0 | 10767 | 10767 | 296070 | 10767 | 3.64 |
| 0 | 14447 | 16059 | 16059 | 16059 | 0 | 10526 | 10526 | 296070 | 10526 | 3.56 |

**Table S2. Genomic inflation values in EWAS of lung DNA methylation of allergen-exposed mice.**

| **Exposure** | **λ** | **Bacon corrected** |
| --- | --- | --- |
| **Blo t 2** | 1.62 | 1.1 |
| **Blo t 13** | 1.61 | 1.23 |
| **Extract** | 1.81 | 1.25 |

λ, genomic inflation factor.

**Table S3. EWAS results for DMRs after exposure to Blo t 2 corrected by BACON.**

**Table S4. EWAS results for DMRs after exposure to Blo t 13 corrected by BACON.**

**Table S5. EWAS results for DMRs after exposure to *B. tropicalis* extract corrected by BACON.**

**Table S6. Ct values of the endogenous control genes across experimental groups**

| Group | Geometric mean *Actb* | Geometric SD *Actb* | Geometric mean *B2m* | Geometric SD *B2m* |
| --- | --- | --- | --- | --- |
| **Saline** | 18.61 | 1.02 | 20.25 | 1.03 |
| **Blo t 2** | 17.74 | 1.03 | 19.10 | 1.03 |
| **Blo t 13** | 18.06 | 1.02 | 19.26 | 1.02 |
| **Extract** | 17.73 | 1.03 | 19.30 | 1.02 |

SD: standard deviation
